# Cognitive Load Measurement Methods for Usability Testing: A Critical Analysis and Framework for Interface Evaluation

**DOI:** 10.1177/00187208261427867

**Published:** 2026-03-18

**Authors:** Ali Darejeh, Nadine Marcus, Gelareh Mohammadi, John Sweller

**Affiliations:** 1 School of Computer Science, Faculty of Engineering, UNSW, Sydney, Australia; 2 School of Education, Faculty of Arts, Design and Architecture, UNSW, Sydney, Australia

**Keywords:** cognitive load theory, usability testing, human–computer interaction, user interface evaluation, cognitive load measurement, EEG, NASA-TLX, dual-task paradigm, performance metrics, eye tracking

## Abstract

**Objective:**

This systematic review evaluates the use of cognitive load measurement methods in usability testing across diverse software interfaces. It provides guidance for researchers and practitioners by proposing a framework for selecting appropriate cognitive load measurement techniques.

**Background:**

Cognitive Load Theory offers insights into software usability by addressing users’ mental effort during task performance. Although cognitive load measurement methods are increasingly used in usability testing, no comprehensive analysis has focused specifically on various software interfaces.

**Method:**

We systematically analysed 87 experimental studies published between 2001 and 2025. Databases searched included IEEE, ACM, ScienceDirect, SpringerLink, and Scopus. Inclusion criteria focused on studies applying cognitive load measurement to usability testing for different types of software interfaces.

**Results:**

Cognitive load measurement methods were categorised as subjective (e.g., NASA-TLX and self-reports) or objective (e.g., EEG, eye-tracking, dual-task paradigms, and physiological sensors). The most frequently used methods were performance measures (19%), NASA-TLX (12%), and eye fixations (11%). Commonly evaluated platforms included websites, virtual reality systems, and productivity tools. Each method’s applicability, strengths, and limitations were identified.

**Conclusion:**

The review synthesises the relative merits of cognitive load measurement methods in usability evaluations and introduces a framework to guide the selection of techniques based on interface type and evaluation goals.

**Application:**

The proposed framework operationalises CLT to support targeted, user-centred usability testing. It facilitates the selection of effective cognitive load measurement strategies, enhancing evaluation accuracy and informing better software design.

## Introduction and Theoretical Background

This paper reviews various cognitive load measurement methods used to evaluate the usability of different types of user interfaces. It also discusses the advantages and disadvantages of each method to identify which techniques are most effective for assessing cognitive load in usability contexts. Cognitive load theory (CLT) was introduced and developed by educational researchers to facilitate learning (Sweller, 1988). It provides instructional guidance based on human cognitive architecture (Sweller, 2025) and our knowledge of evolutionary psychology (Sweller, Ayres, & Kalyuga, 2011; Sweller, Van Merriënboer, & Paas, 2019). Cognitive load is defined as the cognitive resources required to acquire a concept or learn a procedure (Sweller, 1988, 2011). CLT emphasises the limitations of working memory when processing new information during learning or problem solving (Paas Renkl, & Sweller, 2004; Sweller, 1988; Sweller, Ayres, & Kalyuga, 2011). For instance, when users interact with an unfamiliar software interface or learn to operate a new device, they must hold and process multiple elements of information simultaneously, such as icons, navigation paths, and the system structure, which places demand on their working memory and can lead to cognitive overload if the interface is poorly designed.

While CLT was originally developed within educational contexts, its principles extend beyond learning environments and can be applied to other domains involving complex cognitive activity (Engström, Johansson & Östlund, 2005; Huttunen et al., 2011). Interface usability is one of the areas that can significantly benefit from CLT, since the structure of the interface can affect software usability and user’s cognitive load (Hu, Ma, & Chau, 1999; Saadé & Otrakji, 2007). Interface usability can be improved through measuring user’s cognitive load and optimising the interface based on issues that affect user’s cognitive load (Müller et al., 2008).

Usability is one of the central concepts of human–computer interaction (HCI) that is defined as the extent to which a user can work with a product effectively, efficiently, and with satisfaction (Bevan et al., 2015). Usability testing is important to evaluate if the product can be used by target users easily (Nielsen, 1994; Silva et al., 2021). In software interface design, usability depends on how well the system accommodates the characteristics of its target users and the specific tasks they perform. To design highly usable applications, developers must gain a deep understanding of users’ needs, contexts, and interaction behaviours (Sharp, Preece, & Rogers, 2019). Accordingly, cognitive load measurement methods can serve as effective tools for conducting accurate and systematic usability evaluations. This review paper is expected to be relevant to researchers and practitioners interested in cognitive load measurement within user interface contexts. The intended audience includes usability experts, user experience (UX) designers, HCI researchers, and computer scientists who evaluate or design interactive systems. The findings may assist professionals involved in assessing and improving the usability of information systems, e-learning platforms, video games, immersive technologies such as virtual and augmented reality applications, healthcare devices, manufacturing systems, smartphone applications, and websites by guiding their selection of suitable cognitive load measurement methods and interpretation of results.

In the upcoming sections, we delve into the human cognitive architecture and explore the different types of cognitive load. We subsequently provide a detailed description and analysis of various measurement methods for cognitive load, encompassing subjective and objective approaches. These methods are specifically pertinent to the area of usability evaluation for diverse software interfaces. Following that, the discussion section presents a comparative analysis of these measurement methods, evaluating their strengths and limitations. This analysis is followed by the introduction of our proposed framework, which aims to assist usability testers in selecting an appropriate cognitive load measurement method for conducting precise usability evaluations. The framework takes into account various criteria to ensure the selection of the most suitable method.

### Human Cognitive Architecture and Cognitive Load Theory

Based on evolutionary psychology, information processed by the human cognitive system can be divided into biologically primary or secondary categories (Geary, 2008, 2012; Geary & Berch, 2016). The primary category consists of information that over countless generations we have evolved to acquire easily and unconsciously. An example is learning to listen to and speak our native language. Biologically secondary information consists of a category that we need for cultural reasons but require instruction and conscious effort to acquire. Learning to read and write provides an example as does learning to use a computer program. Cognitive load theory is principally concerned with biologically secondary information.

There is a specific cognitive architecture that allows humans to acquire, process, and use biologically secondary information (Sweller, 2025). Within the framework of CLT, information acquisition refers specifically to how learners obtain biologically secondary knowledge that demands deliberate cognitive processing and the engagement of working memory. Such information can be acquired either through individual problem solving, where new knowledge is actively constructed through exploration, or through direct instruction and transmission from others (Geary, 2008; Sweller, 1988; Sweller, van Merriënboer & Paas, 2019). These two modes of acquisition differ from other well-established learning mechanisms, such as observational learning, or probabilistic inference, which are typically associated with biologically primary learning processes that do not impose the same working-memory limitations emphasised in CLT (Bandura & Walters, 1977; Sutton & Barto, 2018).

Once information is acquired, it must be processed within the constraints of our human cognitive architecture. Novel information from the environment is first temporarily held and processed in working memory, which has limited capacity and duration, before being encoded into long-term memory for later retrieval. Once information is stored in long-term memory, it can be retrieved through memory retrieval processes and temporarily held in working memory to guide action appropriate to the current context. For example, when a user interacts with an unfamiliar software interface, they must consciously attend to each step, such as locating menu options, interpreting icons, and remembering procedural sequences, placing a heavy demand on working memory. With repeated use, these actions become automated and integrated into long-term memory, allowing the user to navigate the interface efficiently with minimal cognitive effort.

Although working memory is capacity-limited when processing novel information (typically 7 ± 2 elements; Miller, 1956), these limits can be effectively bypassed when dealing with familiar information that has been automated and stored in long-term memory. Within the CLT framework, this limitation is considered theoretical rather than absolute: when dealing with familiar or automated information stored in long-term memory, working memory is assumed to operate as if its capacity is no longer limited (Paas, Renkl, & Sweller, 2004; Sweller, 2025). In such cases, information is retrieved as integrated “chunks,” reducing the load on working memory (Sweller, 2010). This cognitive architecture provides the foundation for CLT and underpins how mental effort is managed during complex learning and problem-solving tasks.

Beyond this cognitive architecture, it is also important to situate CLT within the broader landscape of psychological constructs concerned with mental effort and performance. In broader cognitive psychology, CLT is closely related to, but distinct from, the construct of Mental Workload. Both address the allocation of limited cognitive resources during task performance, but they differ in theoretical orientation and application. Mental Workload originates from human-factors and ergonomics research and is often operationalised through subjective scales (Cain, 2007; Hart & Staveland, 1988). While both frameworks share methodological overlap in measurement, CLT places stronger emphasis on learning outcomes and schema acquisition, whereas MWL focuses on performance efficiency and operator strain. Understanding their complementarity helps clarify why cognitive load measures are increasingly adopted in usability research that bridges both perspectives.

### Different Types of Cognitive Load

There are two types of cognitive load: intrinsic and extraneous that interact to produce the total cognitive load (Sweller, 2010).

#### Intrinsic Load

Intrinsic cognitive load is defined as the natural complexity level of a specific instructional topic or an entity such as a mathematical concept, software, or a device. It is fixed and cannot be changed, except by altering the entity design or knowledge level of the learner. For example, statistical analysis and 3D modelling applications involve multiple interdependent steps and complex visual–spatial relationships, which inherently increase element interactivity and contribute to a higher intrinsic cognitive load. Intrinsic load is determined by the number of information elements that must be processed simultaneously and the extent to which they interact to achieve a learning goal (Sweller, 1994).

In cognitive load theory, element interactivity is an index to measure the complexity of a learning topic and depends on the learners’ prior knowledge, nature of the materials, and the relationship between the concepts that users should connect and process simultaneously (Chen et al., 2015; Marcus, Cooper & Sweller, 1996; Sweller, 1994, 2010). Elements with low interactivity can be learnt in isolation, with minimal or no reference to other learning elements, for example, learning the features like bold, italic, and underline in MS Excel. Low interactivity elements impose a low working memory load because each element can be processed independently of every other element. However, elements with high interactivity consist of different integrated elements that cannot be learnt in isolation, for example, typing functions in MS Excel or drawing a chart (Sweller, 2010). High levels of element interactivity impose a heavy working memory load because all the elements must be processed simultaneously. Levels of intrinsic cognitive load should be optimised to ensure learning is maximised without overloading working memory.

Although intrinsic load can sometimes appear to be influenced by system design, it fundamentally reflects the inherent complexity of the task or information being processed. For example, interacting with a multi-step data-visualisation tool may impose a high intrinsic load because the user must coordinate several interdependent elements to complete the task. Likewise, even a simple or well-designed interface can impose a high intrinsic load when it represents operations that are conceptually complex in nature. In both cases, the source of difficulty stems from the intrinsic complexity of the material or task requirements rather than from the interface design itself.

#### Extraneous Load

Extraneous cognitive load is related to the difficulty imposed by the method used to present instructional materials or the complexity level of an interface or a device as a result of the way they have been designed. In contrast to intrinsic load, which is tied to the essential complexity of a task or information, extraneous cognitive load stems from preventable design inefficiencies that add unnecessary mental effort beyond what is required by the task at hand. Examples include cluttered layouts, inconsistent iconography, or unclear navigation paths that force users to expend cognitive resources on interface management rather than the task itself. Intrinsic and extraneous load differ in origin: intrinsic load reflects inherent task difficulty, whereas extraneous load arises from avoidable design-induced barriers.

Some instructional procedures increase element interactivity and so unnecessarily increase extraneous load. Common examples of extraneous load include redundant information such as repeated links, menus, or decorative graphics on websites, which are non-essential to the task at hand and may in fact be distracting and consume limited cognitive resources (Bus et al., 2015; Jin, 2012; Lehmann, Hamm, & Seufert, 2019). Split attention is another common type of extraneous load, where users have to split their attention between different screens, windows, or parts of an interface in order to perform a task or understand a website or interface. The user then needs to use cognitive resources to mentally integrate information that is physically separated and that is non-essential to the task at hand (Al-Shehri & Gitsaki, 2010; Sweller, Kalyuga & Ayres, 2011; Schroeder & Cenkci, 2018).

In the context of interface usability, extraneous cognitive load may refer to the design of the user interface, tooltips and software help system that are designed to guide users while working with the interface. In contrast to intrinsic load, which reflects task complexity, extraneous load results from the design and presentation of the interface and instructional materials (Van Merriënboer & Sweller, 2005). For example, websites are generally not difficult in nature, but a poor interface design can make a website difficult to learn, or a good help system that is designed for complex software can make learning the software easier, even if the software is difficult in nature.

Designing an interface based on principles of good design and providing an appropriate help system can enhance learning performance by increasing available working memory resources and reducing extraneous load (Chandler & Sweller, 1991). When the intrinsic load is high, total cognitive load can be decreased by decreasing extraneous load. Learning is enhanced by minimising mental resources that are allocated to deal with a user interface and teaching materials so that working-memory can use its maximum resources to deal with the instructional topic (Sweller, 2010). Therefore, when the nature of the software or the associated interface are cognitively demanding, a good user interface design or an appropriate and comprehensive help system become important ways to decrease extraneous cognitive load.

## Review Methodology

We used the Kitchenham and Charters (2007) methodology, which is a widely adopted approach in computer science and human–computer interaction research. We did not use other frameworks, such as the PRISMA protocol because PRISMA is primarily designed for systematic reviews involving quantitative meta-analyses and intervention-based studies, particularly in health and medical research (Page et al., 2021). In contrast, the present review aimed to qualitatively synthesise methodological patterns and theoretical perspectives on cognitive load measurement in usability evaluation rather than to aggregate statistical outcomes. Therefore, the Kitchenham and Charters approach was more suitable for achieving the objectives of this study, providing a structured yet flexible framework for identifying, categorising, and analysing relevant literature within the context of software usability and cognitive load research. We carried out this review in three main phases: (a) planning of systematic mapping, (b) conducting the review, and (c) reporting the review. Systematic mapping is a structured literature review approach used to classify and summarise existing research within a broad topic area, providing an overview of research trends, gaps, and methodological characteristics rather than quantitative synthesis.

**Figure 1.**
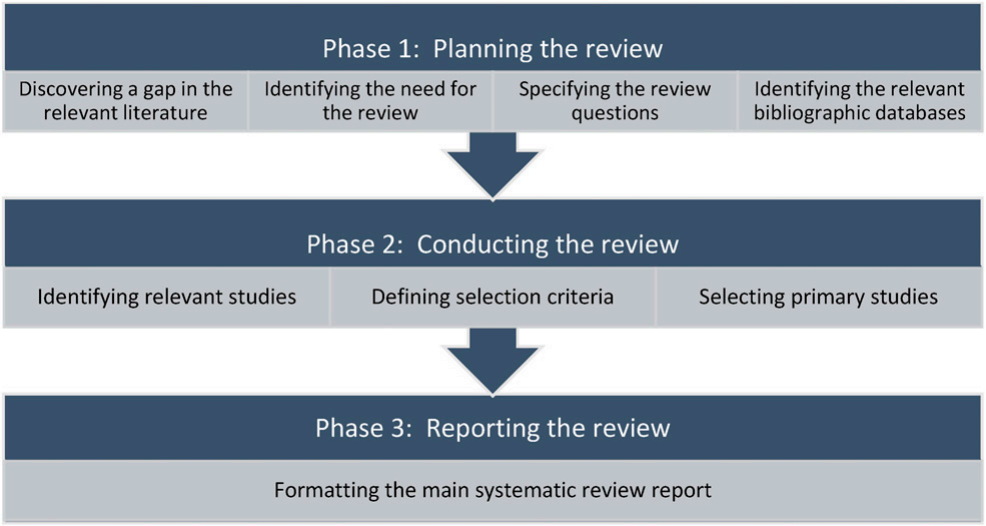
Phases of conducting this systematic review.

The phases of this systematic review and the related activities are shown in Figure 1.

### Phase 1: Planning the Review

#### Discovering a Gap in Relevant Literature

In this step, a comprehensive search was performed on web databases to locate studies relevant to cognitive load measurement methods that are used in the area of evaluating software usability. Although there are a few studies that reviewed different cognitive load measurement methods in the area of computer science, to the best of our knowledge, none have comprehensively listed and compared the full range of cognitive load measurement approaches, including subjective self-report, physiological, behavioural, and performance-based methods, specifically within the context of software usability, nor identified which techniques are most suitable for evaluating the usability of different types of user interfaces.

For example, Duran, Zavgorodniaia and Sorva (2022) conducted a systematic review to identify how CLT has been used across a number of computing education research forums. Frazier, Pitts and McComb (2022) examined how cognitive load is evaluated in studies with a focus on human-automated knowledge work. They compared the studies that evaluated the impacts of automation on cognitive load of operators.

In a recent study, Kosch et al. (2023) conducted a systematic review of cognitive load measurement methods used in HCI. Their focus was on defining and interpreting cognitive workload within HCI and exploring its broader applications in interactive computing systems. While their review covered various cognitive load measurement methods, it provided a broad overview rather than a detailed analysis of their applicability to usability evaluation across different user interfaces. Similarly, Suzuki, Wild, and Scanlon (2024) examined the use of cognitive load measurement methods specifically in the context of augmented reality applications. In contrast, this paper offers a more targeted examination of cognitive load measurement methods, focussing on their advantages and disadvantages in assessing usability across diverse user interfaces within HCI. By narrowing our scope, we aim to provide a practical framework for selecting the most suitable cognitive load measurement techniques for specific interface evaluations.

#### Identifying the Need for a Review

Evaluating the usability issues of computer software, websites, and mobile apps by considering users’ cognitive load is an efficient method as it considers users’ cognitive structure, and it can involve both qualitative and quantitative measures. However, we are unaware of any review that summarises and compares all the possible cognitive load measurement methods that are available and can be used in different areas of software usability.

#### Specifying the Review Questions

The questions we aimed to examine in this review sought to identify and summarise all cognitive load measurement methods that can be used in software usability evaluation. In addition, given the diversity of identified methods, we also considered their validity and practical applicability so that the review contributes more than a descriptive catalogue of methods. The questions are listed below:

• Q1 What are the existing cognitive load measurement methods that have been effectively used in the area of software usability?
• Q2 What are the most common cognitive load measurement methods that can be used in the area of software usability?
• Q3 What types of software have been evaluated by using cognitive load measurement methods?
• Q4 What types of software have been evaluated by using each cognitive load measurement method?
• Q5 What cognitive load measurement methods have been used for evaluating the usability of each type of software?
• Q6 How valid are the identified cognitive load measurement methods, particularly in terms of convergent validity, for evaluating software usability?

#### Identifying the Relevant Bibliographic Databases

In order to answer our questions and find the relevant studies, we tried to select bibliographic databases that cover the majority of journals and conference papers associated with the field of computer science and HCI. The following relevant bibliographic databases were identified: ACM, IEEE, SpringerLink, ScienceDirect, Proquest, Scopus, Wiley Inter Science, and Google Scholar. The search included articles published from 2001 up to February 2025.

### Phase 2: Conducting the Review

This is the main phase of our systematic review with the purpose of selecting related studies as well as synthesising data.

#### Identifying Relevant Studies

In order to locate meaningful papers in bibliographic databases, we conducted a complete search with a combination of five categories of terms (see Table 1). The construction of search terms was based on the steps described by Brereton et al. (2007). Accordingly, Boolean “OR” was used for alternative spellings, synonyms or alternative terms, and Boolean “AND” was applied to connect the main terms. For example, a typical query string used in this review was structured as (‘NASA-TLX’ OR ‘fNIRS’ OR ‘HRV’) AND (‘Cognitive load’ OR ‘CL’) AND (‘Measurement’ OR ‘Method’) AND (‘Usability’ OR ‘Interface usability’) AND (‘Software’ OR ‘Mobile app’).

**Table 1.** Search Keywords.

Term Group	Keywords (OR Within Each Group)	Boolean Connector
Term 1 – Cognitive Load Measures	NASA-TLX OR Electroencephalography (EEG) OR Functional near-infrared spectroscopy (fNIRS) OR Magnetoencephalography (MEG) OR Functional magnetic resonance imaging (fMRI) Or Task completion Or Pupil dilation Or Dual task paradigm method Or Time duration Or Blink rate Or Performance measures Or Mouse dynamics Or Fixation Or Facial Expressions Or Linguistic Features Or Saccades Or Electrodermal Activity (EDA) Or Eye Movement Or Blood pressure Or Heart rate Variability (HRV)	AND
Term 2 – Cognitive Load Concept and Construct	Cognitive load OR CL	AND
Term 3 – Measurement and Assessment Context	Measurement OR Method	AND
Term 4 – Usability and Interface Focus	Usability OR Interface usability	AND
Term 5 – Context of Use/Application Domain	Software OR Mobile app OR Website OR System OR Virtual reality	AND

The terms listed in the Term 1 category represent the set of cognitive load measurement keywords derived from our initial exploratory phase. During this phase, we searched the bibliographic databases listed in Table 2 to compile a comprehensive list of cognitive load measurement methods. After identifying all major and commonly used techniques, we proceeded with the main search phase to locate and include relevant studies for our systematic review.

**Table 2.** Summary of the Search in Bibliographic Databases.

Academic Databases Searched	Scopus	Number of Works Identified
	ScienceDirect	144
	Wiley Inter Science	34
	ACM Digital Library	170
	Springer Database	40
	IEEE	218
	Google scholar	44
Target items	Journal papers	256
	Workshop papers	9
	Conference papers	342
	Book chapters	43
Search applied to	Title	
	Abstract	
	Keywords	
	Content	
Language	Papers written in English	
Publication period	January 2001 up to February 2025	650

Terms 2–3 were used to narrow the search to studies that applied the cognitive load measurement methods identified in Term 1 specifically for measuring cognitive load. Terms 4–5 added an additional layer of criteria, further refining the search to include studies focussing on software usability across different types of applications.

Preliminary test searches conducted without Term 1 retrieved largely overlapping results with fewer unique studies, indicating that the inclusion of known measures enhanced recall without limiting discovery. Additional root terms such as *web*, *internet*, and *online* were also tested during pilot queries but did not substantially expand the result set, as most relevant papers already used the terms “website,” “software,” or “interface” in their metadata.

Two additional search strategies were applied to retrieve the maximum number of relevant papers. The first strategy reviewed the reference list of selected papers to find more related papers. The second strategy involved searching for the authors of the selected studies in Google Scholar to identify additional related research. A summary of the search in bibliographic databases is presented in Table 2.

#### Defining Selection Criteria

In order to select the primary papers, we defined the following criteria based on the purpose of our study.

Inclusion criteria:

(1) Studies that measured users cognitive load to evaluate the usability of any type of software including computer software, websites, mobile applications, virtual reality applications, and video games.
(2) Studies that were published between January 2001 and February 2025.

Exclusion criteria:

(1) Studies that measured cognitive load in another context rather than software.
(2) Non-experimental papers.
(3) Papers available only in the form of an abstract or presentation.
(4) Papers not written in English.

#### Selecting Primary Studies

The titles and abstracts of the retrieved papers were screened according to the predefined inclusion and exclusion criteria. Studies meeting at least one inclusion criterion and none of the exclusion criteria were retained for further review. When eligibility could not be determined based solely on titles and abstracts, full texts were examined. Screening and selection were conducted by two reviewers. Any disagreements were resolved through discussion and consensus, and when consensus could not be reached, a third reviewer was consulted.

The database search across ACM, IEEE, SpringerLink, ScienceDirect, Scopus, Wiley InterScience, ProQuest, and Google Scholar initially yielded 650 records. After removing 35 duplicates, 615 papers remained for screening. Of these, 344 were excluded for being unrelated to usability or cognitive load. The remaining 271 full-text articles were assessed for eligibility based on methodological completeness and relevance to software usability evaluation. Following this assessment, 184 papers were excluded because they did not include a usability evaluation, measured cognitive load outside a software context, or lacked sufficient methodological detail. The final 87 studies met all criteria and were included in the review.

### Phase 3: Reporting the Review

In reporting the results of this study, we used a taxonomy that builds on the dual-dimension framework proposed by Brunken et al. (2003), which has been widely applied in cognitive load research to distinguish measurement methods according to their level of objectivity and causal proximity to cognitive processing. In line with this framework, the reviewed studies were categorised along two dimensions to ensure conceptual consistency and comparability across different types of measures:

(a) The objectivity of the measure (subjective vs. objective) and
(b) The causal relationship between the measure and cognitive load (direct vs. indirect).

The objectivity dimension differentiates between subjective approaches, which rely on self-reported data such as questionnaires, and objective approaches, which depend on behavioural observations, physiological signals, or performance outcomes.

The causal-relations dimension distinguishes between direct and indirect measures of cognitive load according to their proximity to cognitive processing. Direct measures capture indicators that respond immediately to mental activity or cognitive effort, offering fine-grained temporal resolution of users’ mental states during task performance. Examples include self-reported task difficulty or mental effort ratings, neurophysiological methods such as EEG and fNIRS, and the dual-task paradigm, where secondary-task performance directly reflects the cognitive resources consumed by a primary task.

Indirect measures, by contrast, infer cognitive load from its downstream effects or outcomes, such as task accuracy, error rate, completion time, or reaction time. Physiological responses like pupil dilation, HRV, EDA, or facial temperature are also indirect indicators because they can be affected by factors beyond cognitive demand, including emotion or fatigue. Because many indicators overlap conceptually (e.g., arousal or motivation), the distinction between direct and indirect measures is treated pragmatically rather than absolutely.

This structure was selected because it integrates both cognitive-psychology perspectives and human–computer-interaction methodologies, allowing systematic categorisation of diverse approaches ranging from self-reports to neurophysiological recordings. The inclusion of sub-categories such as dual-task, performance, behavioural, linguistic, and physiological measures reflects subsequent methodological developments that operationalise these dimensions in usability contexts (Kosch et al., 2023). Establishing this taxonomy as the organising framework provides conceptual consistency across studies and forms the analytical foundation of the present review. Table 3 summarises this dual-dimension classification of cognitive load measurement methods.

**Table 3.** Classification of Cognitive Load Measurement’s Methods.

Dimension	Category	Measurement Examples	Description
Subjective – Direct	Usability questionnaire	Users rate task difficulty or interface clarity	Captures self-reported experience of difficulty directly tied to task complexity.
Subjective – Indirect	NASA-TLX	Users assess mental effort, frustration, and performance	Reflects perceived workload indirectly related to cognitive load.
Objective – Direct	EEG, fNIRS, dual-task paradigm	Measures cognitive activity or performance interference	Captures immediate resource allocation or neural activation during tasks.
Objective – Indirect	Performance, eye tracking, EDA, HRV, mouse dynamics, linguistic features, facial expressions	Infers load from downstream effects (errors, response time, physiological changes)	Reflects secondary effects of increased load.

Regarding the format used to report the results for each measurement method, we began each section by describing the method’s purpose, usage, and underlying mechanism. This is followed by a summary table presenting the studies that employed that specific method. When a study used multiple methods, it was included only once under the section most closely aligned with its primary focus. For example, a study evaluating the efficiency of the dual-task paradigm appears exclusively under that heading, even if it also used other measures. Tables 4–18 collectively present all 87 studies that met the inclusion criteria of the systematic review and therefore represent a comprehensive account of the reviewed literature rather than illustrative samples.

**Table 4.** Studies That Used Usability Questionnaires to Evaluate Usability.

Studies	Measurement Method	Evaluated Software	Purpose of the Evaluation
Fink, Eisenlauer, and Ertl (2023)	Usability questionnaire	Virtual reality field trip application	Evaluating the usability and cognitive load
Wu, Liu, and Huang (2022)	Usability questionnaire	A block-based visual programming language development tool for learning programming	Evaluating the relationship between usability and cognitive load during learning
Koć-Januchta et al. (2022)	Usability questionnaire, cognitive load questionnaire	An e-learning system for learning biology	Evaluating the relationship between usability and cognitive load during learning

**Table 5.** Studies That Used the NASA-TLX Test to Evaluate Usability.

Studies	Measurement Method	Evaluated Software	Purpose of the Evaluation
Darejeh et al. (2024b)	NASA-TLX	A virtual reality environment for teaching programming	Comparing users’ cognitive load across two different interaction methods
Clarke et al. (2020)	NASA-TLX; interview	Personal health record system	Usability during data entry
Pollack and Pratt (2020)	NASA-TLX	Electronic health records	Evaluating physicians’ cognitive load during clinical prioritisation
Brendle et al. (2020)	NASA-TLX; task performance duration	Surgical navigation system	Evaluating if the system can facilitate spinal fusion surgery
Rockman et al. (2020)	NASA-TLX; usability questionnaire	Cyber operations battlefield application	Performance of a system for teaching cyberspace and electromagnetic activities to military personnel.
Pachunka et al. (2019)	NASA-TLX; interview	Personal health record system	Usability during data entry
Richardson et al. (2019)	NASA-TLX; usability questionnaire	Electronic clinical decision support tool	Overall usability of the system for physicians
Galais, Delmas, and Alonso (2019)	NASA-TLX	Gestural interaction vs gamepad in virtual reality	Comparing cognitive load of users

**Table 6.** Studies That Used EEG Devices to Evaluate Usability.

Studies	Measurement Method	Evaluated Software	Purpose of the Evaluation
Caldiroli et al. (2023)	EEG	Online information-seeking	Measuring cognitive load
Cano et al. (2021)	EEG; usability questionnaire; NASA-TLX	Interactive systems	Comparing the usability of two interactive systems
Plazak et al. (2019)	EEG; NASA-TLX	Image-guided surgery application	Measuring users’ cognitive load
Baig and Kavakli (2018)	EEG; usability questionnaire	Different input methods in AutoCAD	Comparing users’ 3D object drawing performance using speech/gesture vs. keyboard/mouse.
Sengupta et al. (2017)	EEG	Gaze-based virtual keyboards	Finding the efficiency of EEG in measuring usability

**Table 7.** Studies That Used fNIRS Devices to Evaluate Usability.

Studies	Measurement Method	Evaluated Software	Purpose of the Evaluation
Lamb et al. (2018)	fNIRS	Virtual reality’s science education application	Virtual reality’s effect on student’s cognitive load
Kitabata et al. (2017)	fNIRS	Touchscreen devices	Evaluating interface usability
Karim et al. (2012)	fNIRS	Video game	Visualise the load on different parts of the brain
Hirshfield et al. (2011)	fNIRS	Different types of software applications and video games	Measuring usability and users’ cognitive load

**Table 8.** Studies That Used Dual-Task Paradigm to Evaluate Usability.

Studies	Measurement Method	Evaluated Software	Purpose of the Evaluation
Wenk et al. (2023)	Dual-task paradigm; NASA-TLX	Interacting with objects in Virtual Reality and Augmented Reality	Comparing users’ cognitive load while using two different versions
Shelton et al. (2021)	Dual-task paradigm; NASA-TLX	An ambient display (ubiquitous computing sub-class)	Evaluating users’ cognitive load
Na (2021)	Dual-task paradigm; NASA-TLX	Information seeking on the web	Evaluating users’ cognitive load during query reformulation
Lai and McMahan (2020)	Dual-task paradigm	Virtual reality applications	Usability of 3 walking metaphors: Human Joystick, Scaled Walking, Walking-In-Place
King (2019)	Dual-task paradigm Task; performance duration	An email application	Evaluating the usability
Giraud, Thérouanne, and Steiner (2018)	Dual-task paradigm; NASA-TLX	Different versions of a website	Effect of filtering irrelevant, redundant information on usability
Van Nuland (2017)	Dual-task paradigm Test; performance Task; performance duration	Interactive e-learning tool	Comparing users’ cognitive load while using two different versions
Albers (2011)	Dual-task paradigm; NASA-TLX	Two websites	Evaluating the usability
Gwizdka (2010)	Dual-task paradigm	A Wikipedia like website	Evaluating the usability
Schmutz et al. (2009)	Dual-task paradigm; NASA-TLX	Online shopping websites	Evaluating the usability

**Table 9.** Studies That Used Performance Measurement Methods to Evaluate Usability.

Studies	Measurement Method	Evaluated Software	Purpose of the Evaluation
Darejeh et al. (2024a)	Test performance mark; NASA-TLX,	Simulated medical science lab in virtual reality	Evaluating users cognitive load
Holden et al. (2020)	Test performance mark; task performance duration; usability questionnaire	Mobile app to inform elderly users about risks and benefits of medications	Evaluating the usability
Rhodes et al. (2019)	Test performance mark; task performance duration	Different modules of a neurological self-assessment platform	Evaluating the usability
Da Costa et al. (2013)	Test performance mark; task performance duration; usability questionnaire	Online survey tool for self-assessment of diet and physical activity	Evaluating the usability
Huang, Eades, and Hong (2009)	Test performance mark; task performance duration; mental effort questionnaire	Graph visualisations	Effectiveness of different methods of visualisations

**Table 10.** Studies That Used Mouse Dynamics to Evaluate Usability.

Studies	Measurement Method	Evaluated Software	Purpose of the Evaluation
Darejeh, Marcus, and Sweller (2022)	Mouse movement data; test performance mark; task performance duration	MS Access	Evaluating users’ cognitive load
Darejeh, Marcus, and Sweller (2021)	Mouse movement data; test performance mark; task performance duration	MS Access	Evaluating users’ cognitive load
Fuller et al. (2020)	Mouse movement data; test performance mark; task performance duration	Patient safety dashboard	Comparing the usability of two versions
Kortum and Acemyan (2016)	Mouse movement data; test performance mark; usability questionnaire	Software application	Evaluating the usability
Grimes and Valacich (2015)	Mouse movement data; test performance mark; task performance duration	Web applications	Measuring users’ cognitive load
Hibbeln et al. (2014)	Mouse movement data; task performance duration	Online insurance claim forms	Measuring users’ cognitive load
Khawaji et al. (2014)	Mouse movement data; mental effort questionnaire	Chat application	Measuring users’ cognitive load

**Table 11.** Studies That Used Linguistic Features to Evaluate Usability.

Studies	Measurement Method	Evaluated Software	Purpose of the Evaluation
Khawaja, Chen, and Marcus (2014)	Linguistic features; subjective rating questionnaire	Bushfire simulator application	Measuring users’ cognitive load
Khawaja, Chen, and Marcus (2012)	Linguistic features including pronouns	Bushfire simulator application	Measuring collaborative cognitive load
Tuček, Mount, and Abbass (2012)	Linguistic features	Sudoku games	Measuring users’ cognitive load
Oviatt (2006)	Linguistic features; number of errors; task performance duration	Different versions of the same interface for teaching maths	Evaluating the usability

**Table 12.** Studies That Measured Fixations to Evaluate Usability.

Studies	Measurement Method	Evaluated Software	Purpose of the Evaluation
Huang and Zhang (2022)	Eye movements (fixation); usability questionnaire	e-learning	Measuring cognitive load of elderly users
Calvo et al. (2022)	Eye movements (fixation); test performance mark; task performance duration	Climate service visualiser tool	Evaluating usability to redesign the system
Zardari, Hussain, and Arain (2021)	Eye movements (fixation); usability questionnaire; test performance mark; task performance duration	e-learning portal	Evaluating the usability
Guerberof, Moorkens, and Brien (2021)	Eye movements (fixation); test performance mark; task performance duration; satisfaction questionnaire	Microsoft Word	Evaluating the usability
Realpe-Muñoz et al. (2018)	Eye movements (fixation)	E-voting systems	Evaluating users’ cognitive load
Cheng and Wei (2018)	Eye movements (fixation); EEG	Music player software	Optimising the interface
Wang et al. (2014)	Eye movements (fixation); task performance duration	An online shopping website	Users’ visual attention and cognitive load
Chynał et al. (2012)	Eye movements (fixation); task performance duration	A mobile application	Evaluating the usability
Bruneau et al. (2002)	Eye movements (fixation); EDA	Freeserve, AOL, TISCALI, and BTopenworld websites	Evaluating the usability

**Table 13.** Studies That Used Saccades to Evaluate Usability.

Studies	Measurement Method	Evaluated Software	Purpose of the Evaluation
Keskin et al. (2020)	Eye movements (saccades + fixation); EEG; task performance duration	Google map	Evaluating the usability
Ehmke and Wilson (2007)	Eye movements (saccades + fixation)	bbc.co.uk and thetrainline.com	Evaluating the usability
Renshaw et al. (2003)	Eye movements (saccades + fixation); error rate; task performance duration	A management information system	Evaluating users’ cognitive load

**Table 14.** Studies That Used Pupil Dilation Measurement Method to Evaluate Usability.

Studies	Measurement Method	Evaluated Software	Purpose of the Evaluation
Dobhan, Wüllerich, and Röhner (2022)	Eye movements (pupil dilation + fixation); NASA–TLX; test performance mark	Mobile ERP systems	Measuring usability
Majooni, Akhavan, and Offenhuber (2018)	Eye movements (pupil dilation + fixation)	A learning management platform	Measuring usability
Jiang et al. (2018)	Eye movements (pupil dilation); task performance duration; usability questionnaire	Ventilators’ user-interfaces	Evaluating usability

**Table 15.** Studies That Used Blink Rate to Evaluate Usability.

Studies	Measurement Method	Evaluated Software	Purpose of the Evaluation
Li et al. (2024)	Eye movements (blink rate + fixation+); NASA-TLX	2D and 3D user interfaces for parcel sorting in a warehouse setting using augmented reality	Comparing users’ cognitive load
Nagy et al. (2023)	Eye movements (pupil dilation + saccades + blink rate + fixation+); NASA-TLX	In-vehicle information systems	Comparing the usability of two versions
Sevcenko et al. (2023)	Eye movements (pupil dilation + saccades + blink rate + fixation+)	A serious game simulating time-critical emergency situations	Evaluating users’ cognitive load
Derick et al. (2020)	Eye movements (blink rate)	Video games	Evaluating users’ cognitive load
Fowler, Nesbitt, and Canossa (2019)	Eye movements (blink rate + fixation)	Video games	Evaluating users’ cognitive load
Mazur et al. (2019)	Eye movements (blink rate); NASA–TLX; test performance mark; task performance duration	Electronic health record systems	Comparing the usability of two versions

**Table 16.** Studies That Used EDA Devices to Evaluate Usability.

Studies	Measurement Method	Evaluated Software	Purpose of the Evaluation
Barathi et al. (2020)	EDA; interview; test performance mark	Video game	Evaluating users’ cognitive load
Shi et al. (2007)	EDA	Traffic control application	Evaluating users’ cognitive load
Mandryk, Atkins, and Inkpen (2006)	EDA; difficulty questionnaire	Video game	Evaluating users’ engagement and emotion while playing the video game
Nakasone, Prendinger, and Ishizuka (2005)	EDA	Video game	Evaluating users’ engagement and emotion while playing the video game

**Table 17.** Studies That Used HRV Devices to Evaluate Usability.

Studies	Measurement Method	Evaluated Software	Purpose of the Evaluation
Zhou et al. (2020)	HRV, EEG, and EDA	Surgery robotic systems	Evaluating users’ cognitive load
Rajavenkatanarayanan et al. (2020)	HRV and EDA	Robot interfaces	Evaluating users’ cognitive load during robot co-operation
Chan et al. (2020)	HRV, EDA, and interview	Conversational memory coach mobile application	Evaluating users’ cognitive load
Collins et al. (2019)	HRV and EDA	E-learning in virtual reality	Evaluating users’ cognitive load
Gupta et al. (2019)	HRV, EEG, and EDA	Virtual agent within a virtual reality interface	Interaction between cognitive load and users trust
Solovey et al. (2014)	HRV and EDA	In-vehicle user interfaces	Evaluating users’ cognitive load

**Table 18.** Studies That Used Facial Expression.

Studies	Measurement Method	Evaluated Software	Purpose of the Evaluation
Guran, Cojocar, and Dioşan (2020)	Facial expressions	An e-learning application	Evaluating the usability for children
Dargent et al. (2019)	Facial expressions; eye movements (pupil dilation); EDA	Web interfaces	Measuring users’ cognitive load
Xu et al. (2018)	Facial expressions; test performance mark; task performance duration; satisfaction questionnaire	In-vehicle information systems	Evaluating the usability
Machado et al. (2018)	Facial expressions; task performance duration; mouse movement data; test performance mark	Lung auscultation–sound software	Evaluating the usability
Corcoran et al. (2012)	Facial expressions; eye movements (blink rate)	Video games	Evaluating users’ cognitive load
Thüring and Mahlke (2007)	Facial expressions; usability questionnaire; EDA; HRV; test performance mark; task performance duration	Menu and button design of a video Player	Comparing the usability of two different versions

Most of the 87 reviewed studies employed cognitive load measurement methods as practical tools for evaluating user-interface usability rather than as instruments undergoing formal psychometric validation, meaning that validity coefficients were rarely reported. Consequently, validity was assessed through convergent validity, following Campbell and Fiske’s (1959) principle that different methods intended to measure the same construct should yield comparable outcomes.

A method was therefore considered to provide evidence of validity when it consistently revealed similar usability-related trends across studies, indicating cross-study robustness, and when its results aligned with those obtained from additional measurement methods within the same study, such as task-performance data, fixation behaviour, or dual-task outcomes. Alignment across different measurement approaches was interpreted as convergent evidence contributing to construct validity (Cronbach & Meehl, 1955; Messick, 1995a, 1995b). In line with contemporary validity theory (Cook & Beckman, 2006; Cronbach & Meehl, 1955; Messick, 1995a, 1995b), such convergent evidence was treated as a key contributor to construct-level validity. To operationalise this, the review examined convergence across subjective, behavioural, and physiological measures when used together. Cross-method convergence, supported by consistent patterns observed across studies, provided applied evidence of validity and enabled a coherent evaluation of the empirical soundness and interpretability of cognitive load measures within usability research, in alignment with Research Question 6.

## Results

This section presents the findings of the systematic review based on 87 studies examining cognitive load measurement methods in software usability testing. Results are organised by the dimensions of objectivity (subjective vs. objective) and causal proximity (direct vs. indirect). Each subsection summarises the application, advantages, and limitations of the respective methods.

### Subjective Measures

Subjective methods rely on self-report approaches to collect data directly from users after interacting with an interface. Users can rate various factors such as learning, mental effort, or work experience on a 5, 7, or 9-point Likert scale, ranging from 1 (extremely easy) to 9 (extremely difficult). For example, in a software usability test, participants may rate how visually complex or confusing an interface layout appears (a direct measure) or how much mental effort was required to complete a task such as navigating menus or interpreting icons (an indirect measure).

According to a review paper by Van Mierlo et al. (2012), in the context of using cognitive load measurement methods for interface and e-learning system design, subjective techniques assume that users can reliably monitor and report their cognitive processes while working with an interface. These methods are widely used to measure cognitive load as they are easy to conduct, they do not interfere with the primary task and they have proven to be reliable methods to indicate the amount of cognitive load (Ayres, 2006). In order to increase the reliability of self-report methods, using multi-question assessment has been suggested (Gerjets et al., 2009).

#### Subjective Direct Methods

The main direct method of measuring cognitive load is rating the difficulty of interface layout and functionality, after users work with an interface (DeLeeuw & Mayer, 2008; Gerjets et al., 2009; Kalyuga, Chandler, & Sweller, 1999). This scale is very sensitive in identifying differences in the cognitive load of users; however, users’ knowledge, attention, or task difficulty can affect the results as well (Brunken et al., 2003). Some examples of the Likert-scale questions are: ‘I found the difficulty of the interface layout: Easy/Neutral/Difficult’, ‘I found the difficulty of working with feature x: Easy/Neutral/Difficult’, and ‘I found the navigation through the interface pages: Easy/Neutral/Difficult’. See Table 4 for relevant studies that used usability questionnaires.

Studies that employed usability questionnaires, either alone or in combination with cognitive load questionnaires, identified them as effective tools for assessing interface usability and perceived workload. For instance, Koć-Januchta et al. (2022) combined a usability questionnaire with a cognitive load questionnaire to examine the relationship between interface design and users’ mental effort in an e-learning system, demonstrating consistent associations between usability ratings and perceived cognitive load. Across studies, these self-report instruments were used as applied measures of user experience rather than for formal psychometric validation. The consistent correlation between usability ratings and reported mental effort provides applied evidence of their convergent validity as subjective indicators of cognitive load in usability evaluation.

#### Subjective Indirect Methods

In the direct subjective method, the difficulty of the interface layout and functionality are reported; however, in the indirect methods, users report their amount of mental effort devoted in understanding the interface (DeLeeuw & Mayer, 2008; Paas, Renkl, & Sweller, 2003). Several studies have demonstrated that these self-reported mental effort ratings are reliable and valid indicators of cognitive load when appropriately designed and administered, showing consistent correlations with objective performance and physiological measures (Brunken et al., 2003; Paas, Renkl, & Sweller, 2003; Xiao et al., 2005). Most researchers agree that low reported effort corresponds to low cognitive load, whereas higher cognitive load requires users to expend more effort when interacting with the interface (Reed, Burton, & Kelly, 1985). Supporting this assumption, effort and difficulty ratings have been found to correlate significantly (DeLeeuw & Mayer, 2008).

The choice between subjective indirect and direct cognitive load measurement methods depends on the specific research question, and the nature of the cognitive task. Subjective direct cognitive load measurement methods can provide more direct insight into the cognitive load experienced by the individual and may be easier to administer. However, the accuracy of subjective direct methods may be limited by individual differences in the perception of task difficulty and mental effort, as well as by self-report bias, such as participants’ tendencies to underestimate or overestimate their workload due to social desirability, misinterpretation of scale items, or inaccurate introspection (Brunken et al., 2003; Yates, Gough, & Brazil, 2022).

A widely used Likert-scale questionnaire for measuring the mental effort of users after interacting with a software application or website is the NASA Task Load Index (TLX). It is widely adopted because it provides a multidimensional assessment of workload across six factors, mental, physical, and temporal demands, performance, effort, and frustration, offering a more nuanced evaluation than single-item scales. The NASA-TLX has demonstrated strong validity and reliability and cross-cultural robustness, for example, a large-scale Chinese validation study with 1268 mental workers reported both split-half reliability and Cronbach’s α values exceeding 0.80 (Xiao et al., 2005). This instrument has been applied successfully in diverse human–machine environments involving technologies and equipment such as control rooms and laboratory settings (Albers, 2011).

Similar to other subjective methods of measuring cognitive load, NASA-TLX cannot measure cognitive load while users are working with a website or software. The usage of the NASA-TLX occurs after completing each action and relies on users’ opinion of their previous workload. For example, it can be used to evaluate the cognitive load of users after working with different tools of a software application or different parts of a website. However, since users must rely on their memory and remember the process, some details can be forgotten. Also, the default questions of NASA-TLX should be adapted based on the functionality of the software (Pachunka, 2018; Pachunka et al., 2019).

The NASA-TLX test assesses cognitive load based on six scales consisting of: mental demand, temporal demand, physical demand, performance, effort, and frustration. It asks users the following questions:

(1) How much mental effort was required? (Mental Demand);
(2) How much time pressure did you feel? (Temporal Demand);
(3) How much physical effort was required? (Physical Demand);
(4) How hard did you work to finalise the task? (Effort);
(5) How successful were you in completing the task? (Performance);
(6) How disappointed, bored, or annoyed were you while completing the task? (Frustration Level) (Hart & Staveland, 1988; Pandian & Suleri, 2020; Schmutz et al., 2009).

There are many instances of the NASA-TLX test being used to evaluate usability and cognitive load. See Table 5 for relevant studies that used NASA-TLX test alone or in conjunction with the other measurement methods such as usability questionnaires and interviews.

Across multiple usability studies, NASA-TLX has shown strong convergent validity, with scores consistently aligning with behavioural indicators such as task duration and task completion (e.g., Brendle et al., 2020; Galais, Delmas, & Alonso, 2019). Several investigations also combined NASA-TLX with complementary methods, including usability questionnaires and task-performance metrics, producing coherent interpretations of user workload (e.g., Clarke et al., 2020; Pachunka et al., 2019; Richardson et al., 2019; Rockman et al., 2020). This repeated convergence across independent measures provides applied evidence supporting the construct validity of NASA-TLX as a post-test indicator of perceived workload in usability contexts. Although only a small number of studies reported formal validity coefficients (e.g., Xiao et al., 2005), the recurring agreement between NASA-TLX outcomes and behavioural measures supports its interpretive validity for capturing workload demands during software interaction. This evidence reflects the practical value of NASA-TLX in usability evaluation rather than a formal psychometric validation of the instrument.

### Objective Measures

Although subjective measures of cognitive load are widely used by many researchers, objective methods have their own benefits. Objective methods can be categorised into brain activity measures, dual-task-paradigms, performance outcome analysis, behavioural patterns, and physiological measures other than brain activity measures. A major benefit of objective methods such as behavioural patterns and physiological measures is that they can provide a continuous measure of cognitive load that enables researchers to collect and analyse fluctuations in a stream of data over time, in contrast with the subjective self-reported techniques that only provide a few data points at the end of the usability test. They also do not rely on participants’ subjective assessment of their own load.

#### Direct Methods

In this category, use of brain activity measures and dual-task paradigm methods are common measurements.

##### Brain Activity Measures

Brain activity measurement methods use neuroimaging techniques to evaluate cognitive load based on continuous brain signal measurements while users are performing a task. Neuroimaging techniques can measure brain activities when performing cognitive tasks by visualising brain region activation (Anderson et al., 2011; Just et al., 2001). These measurement methods have several benefits including measuring the load continuously with high sensitivity and helping researchers to distinguish stress from mental workload as they can have the same effect on learning performance. However, these measurement methods are quite intrusive for users, require a time-consuming setup, their data analysis is complex (Baldwin & Cisler, 2017) and they are not suitable for routine applications. Different techniques can be used for measuring brain activity and cognitive load including electroencephalography (EEG), functional near infrared spectroscopy (fNIRS), functional magnetic resonance imaging (fMRI), and magnetoencephalography (MEG). Since EEG and fNIRS in comparison with fMRI and MEG have simpler installation processes with portable equipment, they are more common methods.

##### EEG

One of the most popular brain activity measurement methods is EEG that is designed to capture continuous brain activity including alpha, beta, and theta waves. EEG data changes based on cognitive stimuli and working memory load (Anderson et al., 2011). For example, when task difficulty is increased, alpha and theta bands show more activity (Gevins & Smith, 2003). EEG is performed by placing electrodes on the scalp that can measure voltage fluctuations that are related to ionic current within the brain neurons (Schomer & Da Silva, 2012). These sensors are connected via flexible fibre optic cables to a portable base, which allows researchers to measure cognitive load while participants perform a task in either a standing or walking position.

There are many instances of EEG devices being used to evaluate usability and cognitive load. See Table 6 for relevant studies that used EEG alone or in conjunction with the other measurement methods.

Studies that employed EEG to assess cognitive load in usability contexts consistently demonstrated its effectiveness as a direct and sensitive indicator of mental workload. Across the reviewed literature, EEG-based measures were frequently used alongside complementary methods such as NASA-TLX, usability questionnaires, and task-performance outcomes (e.g., Baig & Kavakli, 2018; Cano et al., 2021; Cheng & Wei, 2018; Plazak et al., 2019). Findings showed that variations in EEG frequency bands, particularly changes in alpha, beta, and theta activity, systematically corresponded with differences in task difficulty, interface complexity, and user performance. This alignment between neural indicators and subjective, behavioural, and performance-based measures provides applied evidence of the convergent validity of EEG for evaluating cognitive load in usability testing. Although the reviewed studies primarily applied EEG as an evaluative tool rather than conducting formal psychometric validation, the repeated convergence of EEG outcomes with established cognitive load measures supports its construct validity as a direct physiological method for assessing cognitive demand during interaction with software interfaces.

##### Functional Near-Infrared Spectroscopy (fNIRS)

fNIRS is a portable brain monitoring technology that can record changes in the cerebral blood flow of the brain. fNIRS records brain activity with the use of near-infrared spectroscopy through optical sensors that are placed on the scalp. Similar to EEG, these sensors are connected via flexible cables to a portable base, which allows researchers to measure cognitive load while participants perform a task in either a standing or walking position (Ferrari & Quaresima, 2012). See table 7 for relevant studies that used fNIRS alone or in conjunction with other measurement methods.

Studies that employed fNIRS to measure cognitive load consistently identified it as an effective method for assessing workload and detecting usability-related challenges. All reviewed studies used fNIRS independently to evaluate interface-related cognitive load rather than to establish formal psychometric validity. Across diverse applications, including virtual-reality learning environments, touchscreen systems, and gaming interfaces, fNIRS reliably detected variations in neural activation that corresponded to differences in task difficulty and user performance (Hirshfield et al., 2011; Karim et al., 2012; Kitabata et al., 2017; Lamb et al., 2018). The alignment between fNIRS activation patterns and behavioural indicators provides applied evidence of convergent validity, suggesting that fNIRS functions as a sensitive physiological indicator of cognitive load in usability evaluation, even though existing studies were designed primarily for performance assessment rather than psychometric validation.

##### MEG and fMRI

Several studies have indicated that both these technologies can be used effectively to measure cognitive load. Since no studies as far as we are aware have been used to evaluate interface usability and since bulky equipment would probably prevent such use, these techniques will not be discussed.

##### Dual-Task Paradigm Methods

The dual-task paradigm is a direct, objective method for measuring cognitive load, grounded in the assumption that individuals possess a limited pool of cognitive resources that must be distributed across concurrent tasks (Padilla et al., 2019). The dual-task method can be used with two different approaches. The first approach is adding a secondary task to a primary task to induce a memory load. In this approach the focus is on the primary task, and it is expected that performance in the primary task will be decreased with a dual-task condition in comparison with a single-task condition (Park & Brünken, 2015). For example, the primary task can be opening different pages of a website as fast as possible, and the secondary task can be clicking on a red spot on the screen. Increasing the time duration of opening website pages can be an indicator of increasing cognitive load.

The second approach uses a secondary task to determine the cognitive load imposed by the primary task. Thus, the focus is on the secondary task and based on the amount of load that the primary task imposes, performance of the secondary task will be changed. For example, the primary task can be working with a specific part of an interface and the secondary task can be clicking on a red spot on the screen. Decreasing the number of clicks can be an indicator of increasing cognitive load (Haji et al., 2015; Martin, 2014; Park & Brünken, 2015; Schoor, Bannert, & Brünken, 2012).

Although some scholars argue that dual-task paradigms measure spare attentional capacity rather than cognitive load itself (Park & Brünken, 2015), the two constructs are closely related: as cognitive load increases, the amount of available attentional capacity decreases. Hence, secondary-task performance provides an immediate behavioural indicator of how cognitive resources are allocated during task execution. While the dual-task paradigm may be viewed as indirectly reflecting cognitive load, in this review it is classified as a direct method because it captures real-time performance outcomes that arise from immediate cognitive processing demands.

For usability testing with the use of the dual-task paradigm method, the primary task is commonly working or learning with the interface, and the secondary task is a visual observation task such as remembering a letter or a word, asking participants to press a keyboard’s key or move the mouse and click a button based on changing the colour of a small window or the appearance a letter or a shape on the screen (DeLeeuw and Mayer, 2008; Lai & McMahan, 2020; Schmutz et al., 2009; Van Nuland, 2017).

One of the most commonly used dual-task methods for measuring users’ cognitive load while interacting with an interface is the Detection Response Task (DRT), in which a secondary manual or tactile response, such as a tapping action, is used to detect changes in attention and cognitive demand (Albers, 2011; Innes et al., 2021; Stojmenova & Sodnik, 2018). In the tapping action method, users are asked to tap (one tap per second) with either their hand or foot while working with a website and the evaluator counts the number of taps by recording a video, recording the tapping sound, or connecting some hardware to the hands or foot of the users to count the number of taps automatically (Olive, 2004). Tapping is a secondary task to the main task which is working with the website and puts an extra load on users. Based on the difficulty of different parts of the website, the number of taps can be either increased or decreased which can be an indicator of increasing or decreasing cognitive load.

For example, to evaluate the cognitive load of users while working with a website, we can use the tapping method by asking users to use one of their hands to control the mouse and the other hand to tap on the desk. If users face any usability issue such as an inability to find the desired feature, it is expected that the number of taps will be decreased as they should expend more mental effort to work with the website. After finding the desired feature, the number of taps should return to the normal baseline rate (Miyake, Onishi, & Poppel, 2004).

Since the dual-task paradigm methods such as the tapping method, measure cognitive load continuously, they can reveal different types of usability issues such as those that cannot be measured with the other methods of measuring cognitive load such as difficulty scale questionnaires that only measure cognitive load at the conclusion of the task. It can even help to find minor usability issues that may not significantly overload users, however, discovering them can help to optimise the design (Albers, 2011). The main drawback of dual-task methods is that the secondary task may influence participants’ performance on the primary task (Van Mierlo et al., 2012), and so some researchers prefer not to use it. See Table 8 for relevant studies that used the dual-task paradigm alone or in conjunction with other measurement methods.

Most of the studies summarised in Table 7 indicated that the dual-task paradigm is an effective method for measuring cognitive load in usability contexts. Several investigations combined the dual-task paradigm with subjective measures such as NASA-TLX (e.g., Albers, 2011; Giraud, Thérouanne & Steiner, 2018; Na, 2021; Schmutz et al., 2009; Shelton et al., 2021; Wenk et al., 2023), while others integrated behavioural indicators such as task completion time and error rates (e.g., King, 2019; Van Nuland, 2017). Across these studies, convergence between subjective (NASA-TLX) and behavioural (dual-task and performance outcomes) measures was consistently observed, providing applied evidence of the convergent validity of the dual-task paradigm for assessing usability-related cognitive load. Two studies (Schmutz et al., 2009; Van Nuland, 2017) did not report significant differences in cognitive load, suggesting that the sensitivity of the dual-task approach may vary depending on task characteristics and experimental design.

#### Indirect Methods

In this category, performance measures, behavioural measures, and physiological measures are the common measurement methods.

##### Performance Measures

One of the most common methods of measuring cognitive load is analysing performance outcomes by calculating correct answers, error rates, and the time duration of performing a task. This method is indirect because it depends on mental processing speed and retrieval which can be affected by cognitive load. In order to compare the usability of two versions of an interface with the use of this method, the same tasks should be assigned to users while they are working with the interfaces and based on the number of correct answers, mistakes, or time duration, a performance mark is calculated. Since the tasks are the same, we can expect that the differences in performance outcomes are a reflection of mental load that is induced by the interface design (Antonenko & Niederhauser, 2010; Brunken et al., 2003; DeLeeuw & Mayer, 2008; Mayer, 2001; Paas, Ayres, & Pachman, 2008; Van Mierlo et al., 2012). For example, in order to evaluate the usability of the drawing chart feature of Microsoft Excel compared to the same feature using Apple Numbers, the task completion rate, and error rate of both drawing chart features can be measured and compared.

A key advantage of performance measures is their objectivity and ease of collection, as they do not rely on users’ self-assessment or specialised equipment (DeLeeuw & Mayer, 2008). They are particularly useful for detecting observable consequences of excessive cognitive load, such as slower task completion or increased errors (Antonenko & Niederhauser, 2010; Van Mierlo et al., 2012). However, their major limitation is sensitivity: performance can be affected by factors unrelated to cognitive load, such as prior experience, motivation, fatigue, or task familiarity, making it difficult to isolate cognitive load as the sole cause of performance variation (Paas, Ayres, & Pachman, 2008; Zheng, 2017). Moreover, very high or very low performance may reflect underload (task too easy) or overload (task too demanding), meaning that optimal interpretations often require triangulation with subjective or physiological measures to ensure accurate conclusions (Antonenko & Niederhauser, 2010; Brunken et al., 2003).

Time-on-task is another performance measurement factor that can be calculated based on the amount of time that users spend completing different tasks while working with different elements of an interface (Brunken et al., 2003; Cranford et al., 2014). Extended time spent on a specific part of an interface may indicate suboptimal cognitive load, which can reflect either overload, when users struggle with excessive task demands, or underload, when tasks fail to maintain engagement and attention (Antonenko & Niederhauser, 2010; Brunken et al., 2003; Khawaja, Ruis, & Chen, 2007). For example, in the context of multimedia systems, navigation speed and errors that increase the time duration of the target task completion can be considered to be a result of cognitive load (Astleitner & Leutner, 1996; Yin et al., 2008). See Table 9 for relevant studies that used the performance measurement method alone or in conjunction with other measurement methods.

Overall, the findings indicate that lower error rates, higher test-performance scores, and shorter task durations function as practical indicators of reduced cognitive load and greater usability. In several studies, performance measures were used alongside self-reported tools such as NASA-TLX or usability questionnaires (e.g., Da Costa et al., 2013; Darejeh et al., 2024a, 2024b; Holden et al., 2020; Huang, Eades & Hong, 2009), and the results across these approaches consistently revealed similar cognitive load trends. This convergence between performance-based and subjective indicators provides applied evidence of the convergent validity of performance measures for evaluating cognitive load in usability contexts, even though most investigations aimed to assess interface performance rather than to conduct formal psychometric validation.

##### Behavioural Measures

Behavioural measurement methods evaluate cognitive load based on users’ behaviour while they are performing a task with a user interface. The most common behavioural measures that are used for usability purposes are evaluating mouse dynamics and linguistic features.

###### Mouse Dynamics

Mouse dynamics are one of the behavioural measures of cognitive load that are supported based on our human motor system. Mouse dynamics rely on different mouse movement attributes including speed, direction, action, distance, and time (Ahmed & Traore, 2007). Based on these attributes, higher level features such as acceleration, angular velocity, scroll wheel activity, clicks/double clicks, drag-and-drop operations, point-and-click operations, and silence can be measured (Ahmed & Traore, 2007; Gamboa & Fred 2004; Hocquet et al., 2004; Jorgensen & Yu 2011; Pusara & Brodley, 2004). These features are aggregated and measured in distinct time periods for analysis (Grimes & Valacich, 2015). There are different applications such as Mouse Tracker and Mousotron that can be used to measure mouse movement data.

Several studies showed that mouse dynamic attributes decrease with increasing cognitive load, since users have less resources to perform the tasks as load increases and so have less resources left to move the mouse (Grimes & Valacich, 2015; Khawaji et al., 2014; Rheem, Verma, & Becker, 2018). In particular, if the mouse movements are redundant to the task at hand, by increasing cognitive load the movement will be decreased. However, if the mouse movement is used to complete the main tasks, the movements may increase when increasing the complexity level of the task as participants require more effort to find the solution and complete the task, and the mouse movements may support this goal. Whether mouse movement increases or decreases as cognitive load changes will depend on the purpose of the movements to the task at hand. See Table 10 for relevant studies that used mouse dynamics in conjunction with other measurement methods.

Mouse-dynamic measures have been recognised as an effective method for assessing cognitive load and identifying usability-related difficulties. Across the reviewed studies, mouse-movement indicators were analysed alongside complementary measures such as task-performance duration, test-performance scores, usability questionnaires, and mental effort ratings (e.g., Darejeh, Marcus & Sweller, 2021, 2022; Fuller et al., 2020; Grimes & Valacich, 2015; Hibbeln et al., 2014; Khawaji et al., 2014; Kortum & Acemyan, 2016). Findings consistently showed that mouse-movement characteristics—such as speed, distance, and acceleration—varied systematically with task difficulty and aligned with subjective and performance-based indicators of cognitive load. Although mouse-dynamics were primarily applied to evaluate interface usability rather than to establish psychometric validity, the convergence observed across multiple measurement methods provides applied evidence of the convergent validity of mouse-dynamic measures for detecting cognitive load variations during interactive tasks.

###### Linguistic Features

One of the indirect subjective methods of measuring cognitive load that can be used in evaluating usability is analysing the complexity level of users’ spoken language including sentence length, number of words, punctuations, syllables, use of pauses, repetitive words, corrections, complexity, and the comprehensibility level of the words. Linguistic complexity is measured by two major factors known as syntactic complexity and semantic difficulty. Syntactic complexity observes the sentence length, which is the best indicator of language complexity. Semantic difficulty analyses the use of words, their lengths (syllables and letters) and their structure (Lennon & Burdick, 2004).

Different studies showed that there will be different linguistic patterns based on the complexity level of the task and the associated cognitive load. Some have indicated in high-load situations, participants’ speech rate, amplitude, speech energy, and variability will be increased (Brenner et al., 1985; Lively et al., 1993). Others have reported peak intonation and pitch range patterns (Kettebekov, 2004; Lively et al., 1993) and pitch variability (Wood et al., 2004) are related to high cognitive load. Also, by increasing the cognitive load level, participants may use significantly longer sentences with more complex structures that can make comprehension more difficult (Khawaja, Chen & Marcus, 2014). Khawaja, Chen and Marcus (2012) also found that as the task complexity increased, so teams collaborate more and their language patterns change as reflected by an increase in speech, use of longer sentences, more disagreement, and using more plural pronouns such as “we” and “they.”

Across these studies, the recording and analysis of language varied: some examined spoken utterances captured through audio recordings (Khawaja, Chen & Marcus, 2014; Oviatt, 2006), whereas others analysed team dialogues or typed linguistic inputs within interactive or simulation-based environments (Khawaja, Chen & Marcus, 2012; Tuček, Mount & Abbass, 2012). These methodological differences partly explain the variation in reported linguistic indicators of cognitive load. Since linguistic methods can measure the load in real time, it can be an efficient method to find different usability issues, especially if software users need to communicate and speak with each other. See Table 11 for relevant studies that used linguistic features alone or in conjunction with the other measurement methods.

The reviewed studies indicate that linguistic features can serve as effective indicators of cognitive load and usability. Linguistic analysis was used either independently or in conjunction with subjective rating questionnaires or performance measures (e.g., Khawaja, Chen & Marcus, 2012, 2014; Oviatt, 2006; Tuček, Mount & Abbass, 2012). Findings consistently showed that increases in task difficulty were associated with reduced speech intensity, higher pitch, and simpler sentence structures, reflecting the diversion of working-memory resources toward task processing. In collaborative contexts, greater cognitive load corresponded with more tightly coupled speech patterns and shared linguistic structures among participants. Although these studies primarily applied linguistic analysis to evaluate user performance rather than to establish formal psychometric validity, the consistent convergence with subjective and other behavioural indicators provides applied evidence of the convergent validity of linguistic features as markers of cognitive load in usability evaluation.

###### Eye Movements

Eye movement can be simply measured with the use of cameras and eye tracking devices without the need to attach anything to users that can make it uncomfortable for them and decrease reliability of the results. Different studies have proved the efficiency of measuring eye movements in the process of usability testing and designing adaptive e-learning systems (El Haddioui, 2019). Some researchers found that when self-report questionnaires cannot show an accurate result in the usability evaluation, eye tracking methods can be used as an alternative and reliable solution (Schiessl et al., 2003; Wang et al., 2020).

Compared to EEG or fNIRS, which capture direct neural activity associated with cognitive processing, eye-movement measures provide an indirect but highly interpretable reflection of visual attention and information-processing strategies. While EEG and fNIRS offer higher temporal precision and sensitivity to instantaneous cognitive fluctuations, eye-tracking methods are less intrusive, easier to implement in usability studies, and can reveal how users allocate attention across interface elements in real time. Accordingly, eye-movement data can be used in conjunction with EEG to triangulate cognitive load findings and enhance interpretive validity (Beatty, 1982; Baldwin & Cisler, 2017; Hess & Polt, 1964).

Eye movement measures can be categorised into behavioural (voluntary) including fixations and saccades and physiological (involuntary) including pupil dilation and blink rate that will be discussed in the physiological measures section (Chen et al., 2016; Pfleging et al., 2016; Rudmann, McConkie, & Zheng, 2003).

Fixation is a behavioural (voluntary) eye movement measurement which refers to a situation when the eyes remains still over a period of time, from 200 milliseconds to several seconds, focussed onto an area of interest (AOI). In this measurement, the interface element that is relevant for the current cognitive activity is identified based on the gaze direction (Rudmann, McConkie, & Zheng, 2003). Increasing the fixation duration on an interface element can show that users needed more attention as the understanding of the interface was difficult for them. It can be an indication of increased processing in working memory and high cognitive load (Chen et al., 2011). See Table 12 for relevant studies that used the fixations measurement method alone or in conjunction with other measurement methods.

The reviewed studies indicate that fixation-based eye-tracking measures are effective indicators of cognitive load and usability. Across investigations, fixation metrics were frequently analysed alongside other measures such as usability questionnaires (Huang & Zhang, 2022; Zardari, Hussain & Arain, 2021), task-performance duration (Calvo et al., 2022; Chynał et al., 2012; Wang et al., 2014; Zardari, Hussain & Arain, 2021), test-performance scores (Calvo et al., 2022; Guerberof, Moorkens & Brien, 2021; Zardari, Hussain & Arain, 2021), EEG (Cheng & Wei, 2018), and EDA (Bruneau et al., 2002). Findings consistently showed that fixation duration and frequency increased with higher task complexity and reduced usability, mirroring patterns observed in error rates and task-completion times. This alignment between visual-attention metrics and subjective, behavioural, and physiological indicators provides applied evidence of the convergent validity of fixation-based measures for evaluating cognitive load. Although most studies focused on applied usability assessment rather than psychometric validation, the consistent fixation-based findings across diverse interfaces underscore their robustness as indicators of attentional demand in usability contexts.

Another behavioural (voluntary) eye movement measurement involves the use of a saccade, which refers to rapid eye shifts between two locations (from one fixation to another) that reflect users’ visual search behaviour and attentional shifts. The most common measurement method for saccades is observing the patterns and velocity of scan paths, defined as the number and duration of gaze fixations across different AOIs. In usability contexts, increasing saccade velocity or frequency indicates higher cognitive load because users must exert greater mental effort to locate relevant interface elements, suggesting inefficiencies in visual layout or information organisation. When cognitive processing is smooth and the interface supports user expectations, saccades tend to be shorter and more targeted; when users struggle to find information, they become faster, more frequent, and less predictable. Conversely, a decrease in saccade velocity can be an indication of tiredness or reduced attentional engagement (Barrios et al., 2004; Chen et al., 2011). See Table 13 for relevant studies that used the saccades measurement method alone or in conjunction with other measurement methods.

In the reviewed studies, saccade-based eye-movement measures were recognised as effective indicators of cognitive load and usability. These measures were frequently examined alongside other indicators such as fixation metrics, task-performance duration, error rates, and EEG (Ehmke & Wilson, 2007; Keskin et al., 2020; Renshaw et al., 2003). Across these investigations, increased task complexity and reduced usability were associated with shorter, more frequent saccades and higher fixation counts, reflecting greater visual-search effort. The convergence between saccadic behaviour, performance outcomes, and physiological responses provides applied evidence of the convergent validity of saccade-based measures for detecting cognitive load variations, even though these studies primarily focused on interface evaluation rather than formal psychometric validation.

##### Physiological Measures

Physiological measures are a group of cognitive load measurement methods that are based on brain activities and our physiological reactions. By increasing mental processing, cortical activity causes a small nervous response within the body that can affect pupil dilation, blink rate, heart rate, blood pressure, facial muscles, and electrodermal activity (Zagermann, Pfeil & Reiterer, 2016).

###### Pupil Dilation and Blink Rate

In the category of physiological cognitive load measures, analysing pupil dilation and blink rate are two of the most reliable approaches and are used by many, as they can be simply measured with the use of cameras and eye tracking devices without the need to attach anything to users that can make it uncomfortable for them and decrease reliability of the results.

As explained in the previous section, eye movement measures can be categorised into behavioural (voluntary) including fixations and saccades and physiological (involuntary) including pupil dilation and blink rate (Chen et al., 2016; Pfleging et al., 2016; Rudmann, McConkie, & Zheng, 2003). In the next sections the physiological measures of eye movement are discussed.

Pupil dilation is a physiological (involuntary) eye movement measurement that is affected by cognitive processes. With increased cognitive load, users’ pupils dilate and by decreasing the difficulty level of the task or towards the end of a task, pupils diameter size decreases (Chen et al., 2011; Klingner, Kumar, & Hanrahan, 2008; Porta, Ricotti, & Perez, 2012; Rafiqi et al., 2015; Rudmann, McConkie, & Zheng, 2003; Zagermann, Pfeil, & Reiterer, 2016). However, it should be considered that in addition to cognitive processes, pupils diameter size can be changed based on the brightness of the environment. Pupils’ diameter size increases when the environment becomes darker to obtain more light and decreases when the environment becomes brighter. Therefore, controlling the brightness of the environment and display brightness are critical factors that can affect pupils’ size and affect the reliability of the usability test results. In order to get more accurate results when testing a user interface based on pupils’ size, the calibration value for the display brightness should be subtracted from the measured pupils’ size (Liu, Zheng, & Zhou, 2019). See Table 14 for relevant studies that used pupil dilation measurement methods.

The reviewed studies demonstrate that pupil-dilation measures are effective indicators of cognitive load and usability. Pupil metrics were frequently analysed alongside other measures such as fixation behaviour, task-performance outcomes, usability questionnaires, and NASA-TLX ratings (Dobhan, Wüllerich & Röhner, 2022; Jiang et al., 2018; Majooni, Akhavan & Offenhuber, 2018). Across these investigations, greater task difficulty and reduced interface usability were consistently associated with increased pupil dilation, longer fixation durations, and higher self-reported workload scores. This alignment between physiological, behavioural, and subjective indicators provides applied evidence of the convergent validity of pupil-dilation measures for assessing cognitive load in usability evaluation, even though the reviewed studies focused primarily on applied interface assessment rather than formal psychometric validation.

Another physiological (involuntary) eye movement measurement is rate and latency of blinking which can show the attention of users. A high blink latency and a low blink rate are an indication of a high mental load (Chen et al., 2011). When users need to process the usage of an interface element that is not easily comprehensible for them and they need to devote more attention to a task, their blink rate will decrease. Therefore, the blink rate is an indication of increasing cognitive load in the area of human computer interaction (Joseph & Murugesh, 2020). See Table 15 for relevant studies that used the blink rate.

The reviewed studies indicate that blink-rate measures are effective indicators of cognitive load and usability. Blink-rate analysis was frequently conducted alongside other physiological and subjective measures such as fixation behaviour (Fowler, Nesbitt & Canossa, 2019; Li et al., 2024; Nagy et al., 2023; Sevcenko et al., 2023), pupil dilation and saccades (Nagy et al., 2023; Sevcenko et al., 2023), NASA-TLX ratings (Li et al., 2024; Mazur et al., 2019; Nagy et al., 2023), and performance outcomes such as task duration and test-score accuracy (Mazur et al., 2019). Across these investigations, higher cognitive load and reduced usability were consistently associated with decreased blink frequency and increased fixation duration, reflecting sustained visual attention and mental effort. The convergence between blink-rate patterns and physiological, behavioural, and self-reported indicators provides applied evidence of the convergent validity of blink-rate measures for assessing cognitive load in usability studies, even though most investigations were designed for applied interface evaluation rather than formal psychometric validation.

When measuring cognitive load using eye movement measurement methods, there are three factors including individual interaction, social interaction, and environmental factors that should be taken into account (Jetter, Reiterer, & Geyer, 2014).

Individual interaction describes the way users interact with a system interface including menus, content, and tools. Based on the system design and the activity that users perform with the system, they will be required to focus on a specific part of the interface which can influence users’ fixations. Thus, interface layout and the visual presentation of content can also influence users’ fixation location, duration, and rate without changing their cognitive load. Also, the interface structure as well as the task at hand can influence saccadic eye movements including length, velocity, and angle of saccades (Zheng, 2017). For example, while users perform a visual analytic task in spreadsheet applications, they need to switch between different tools, worksheets, and charts which can affect fixation and saccadic eye movements. Another example is the difference between eye movements when users are searching for information by reading a text versus checking an image.

The other factor that can influence saccadic eye movements and fixations without affecting users’ cognitive load is the input and output technologies including mouse, keyboard, and touch screen displays that are used to interact with the interface. Input devices might require users to fixate on the input in addition to the visual information on a display (Gao & Sun, 2015). For example, graphic designers often need to switch between desktop displays, and digital pen and tablet to draw an artwork (Keim et al., 2008). Input and output devices can also affect pupil dilation and blinking behaviour as the luminance of the devices such as mobile phones, tablets, and laptops can be changed frequently based on the brightness of the environment (Pfleging et al., 2016). Thus, in terms of individual interaction, it is important to consider the software content, the task at hand, and the input and output devices as additional elements that can influence eye movements without changing cognitive load.

Social interaction describes the social aspects that influence the way we collaborate with the use of the system. Some systems such as online games, telecommunications applications, and e-learning platforms are inherently social, as users need to work with the system in groups and have communication and perform coordination activities with the other users. Depending on the activity, the number of users that interact with each other, and social roles of users, fixations and saccadic eye movements can be affected without changing cognitive load (Zagermann, Pfeil, & Reiterer, 2016). Switching focus between different users and interactive devices is exhausting for the eyes and consequently it can increase blinking rates and the velocity of eye movement as well as fixations and saccades. Therefore, the interference of eye movements through communication with the other users should be considered in order to increase the reliability of the usability test results when they are measured using eye movements (Zagermann, Pfeil, & Reiterer, 2016).

Environmental factors describe the characteristics of the physical environment such as the office, lab, class, or conference room where users interact with the system. Since each environment has its own characteristics such as temperature, humidity, and luminance with different types of furniture, equipment, and devices, users’ attention and consequently eye movements can be changed during the usability test without changing cognitive load. Thus, during a usability test the fixations should be evaluated with respect to the physical surroundings. Also, the environmental characteristics can exhaust the eye and increase blinking. For example, the environmental luminance has the highest impact on pupil size. Therefore, environmental factors should be taken into account when conducting a usability test using an eye movement measurement method as it can interfere with eye tracking measurements in several different ways (Chen et al., 2016).

###### Electrodermal Activity (EDA)

EDA is one of the characteristics of the human body that causes continuous changes in the electrical properties of the skin (Boucsein, 2012; Critchley, 2002). EDA theory indicates that skin resistance varies based on the state of sweat glands in the skin and the sympathetic nervous system is responsible in controlling sweating (Martini & Bartholomew, 2001). By arousing the sympathetic nervous system, the activity of sweat glands is increased, enhancing skin conductance. Therefore, skin conductance can be measured as an indication of physiological arousal (Carlson, 2013). In other words, based on environmental factors and a person’s cognitive state, emotional arousal changes can increase sweat gland activity and consequently lead to an increase in skin conductance. It should be noted that the EDA signal is representative of the intensity of an emotion but not emotion type.

In applied usability testing, EDA is measured by attaching very thin sensors around the fingers or wrist with the sensors sending the signals wirelessly to a computer, then the computer quantifies moments of significant increased arousal (iMotions, 2021). For example, a spike in conductance while a user interacts with a complex or confusing interface element may indicate an increase in cognitive or emotional load at that moment. This allows researchers to align EDA data with specific user interactions, helping identify which interface components contribute to elevated cognitive demand.

Due to the simplicity of the EDA measurement method, its accuracy in detecting changes in human mental states, and the straightforward procedures for quantifying EDA data, it is a suitable technique for measuring cognitive load during usability evaluation (Georges et al., 2017; Nourbakhsh et al., 2017; Veilleux et al., 2020). There are two other commonly used terminologies for EDA, namely, galvanic skin response (GSR) and skin conductance response (SCR) that fall under the umbrella term of EDA, and they all essentially refer to the same concept. In fact, EDA has been known as GSR and SCR historically. In the recent studies, researchers mostly used the acronym EDA, however, in older studies either GSR or SCR were used (Boucsein, 2012). See Table 16 for relevant studies that used EDA.

The reviewed studies demonstrate that EDA measures are effective indicators of cognitive load and usability. EDA was employed either independently or alongside other indicators such as interviews and performance measures (Barathi et al., 2020) or subjective questionnaires assessing perceived task difficulty (Mandryk, Atkins & Inkpen, 2006). Other investigations applied EDA alone to capture physiological arousal associated with increased mental effort (Nakasone, Prendinger & Ishizuka, 2005; Shi et al., 2007). Across these studies, higher task complexity or engagement consistently produced elevated skin-conductance responses, reflecting increased cognitive and emotional activation. The convergence between EDA trends and subjective or behavioural indicators provides applied evidence of the convergent validity of EDA as a physiological measure of cognitive load in usability contexts, even though most studies were designed for applied interface evaluation rather than formal psychometric validation.

###### Heart Rate Variability (HRV) and Blood Pressure

HRV is a measure of variation in the time interval between heartbeats, and it is an indication of changes in blood pressure and mental stress that are controlled by the autonomic nervous system (ANS) and hypothalamus in the brain (Buccelletti et al., 2009; Solhjoo et al., 2019). By increasing cognitive load, both sympathetic and parasympathetic components of the ANS increase, that impact the HRV (Goldstein et al., 2011; Solhjoo et al., 2019). The relationship between HRV and cognitive load is indirect: as cognitive load increases, heart rate, and blood pressure tend to rise, which in turn leads to a reduction in HRV (Ayres et al., 2021; Hjortskov et al., 2004). It should be noted that HRV is a more accurate and sensitive measure of cognitive load compared to heart rate or blood pressure alone, since HRV reflects the central pathway in the cardiovascular mechanism; however, the blood pressure is more influenced by the working muscle conditions (Hjortskov et al., 2004).

HRV is more successful with short-duration basic tasks such as binary decision tasks and to measure large differences in cognitive load such as differences between mentally active and mentally inactive periods (Paas, van Merriënboer & Adam, 1994). Based on several studies, using HRV in longer-lasting learning tasks can decrease the validity and sensitivity of the results (Ayres et al., 2021). Therefore, HRV is more appropriate to evaluate the location of a specific component of the interface that only has one step rather than evaluating a long task with the use of the interface.

HRV can be measured with the use of electrocardiogram (ECG), blood pressure, ballistocardiogram, and photoplethysmography (Bruser et al., 2011; Brüser et al., 2012). In order to evaluate cognitive load, ECG is the most common approach of measuring HRV carried out by placing electrodes on the chest skin (Forte, Favieri & Casagrande, 2019).

Most of the studies that evaluated the efficiency of the HRV method in measuring cognitive load in the context of usability testing, used HRV with a combination of other approaches such as EDA and EEG. See Table 17 for relevant studies that used HRV alone or in conjunction with other measurement methods.

The reviewed studies demonstrate that HRV measures, particularly when combined with other physiological indicators, are effective tools for assessing cognitive load and usability. HRV was consistently evaluated alongside EDA (Chan et al., 2020; Collins et al., 2019; Rajavenkatanarayanan et al., 2020; Solovey et al., 2014), EEG (Gupta et al., 2019; Zhou et al., 2020), and subjective feedback (Chan et al., 2020). Across these investigations, increased task difficulty and higher cognitive demands were associated with reduced HRV and elevated EDA or EEG activity, reflecting heightened physiological arousal and mental effort. The convergence across multiple physiological channels provides applied evidence of the convergent validity of HRV as a complementary indicator of cognitive load in usability evaluation.

The reason HRV has not been used alone as a measure of cognitive load is that HRV alone may not provide a complete picture of cognitive load. While changes in HRV have been linked to cognitive load, they can also be influenced by other factors, such as physical activity, stress, or emotions. Moreover, different types of cognitive tasks may elicit different patterns of HRV, making it difficult to interpret HRV data without additional measures. Therefore, HRV is often used in conjunction with other cognitive load measures to provide a more comprehensive assessment of cognitive load (Bong et al., 2016).

###### Facial Expressions

Measuring facial expression could identify the mental load based on micro-movements of facial muscles, such as frowning, which are an indication of the emotional state of an individual (Cacioppo et al., 1986). Both positive and negative emotions lead to different changes in the face muscles such as changing the form of mouth, eyebrows, and cheeks. In order to measure facial expression, the facial movements are recorded with a regular high-quality camera and the start, duration, and end of each facial movement are taken into account (Freitas-Magalhães, 2013). Commonly an angry face indicates high mental load, but neutral and happy faces indicate low mental load (Johanssen, Bernius & Bruegge, 2019; Pecchinenda & Petrucci, 2016). These interpretations are typically made within the same participant, by comparing moments of higher versus lower arousal during task performance, rather than between different participants.

One of the issues in using facial expressions compared with the other measurement methods of cognitive load such as physiological and task performance is that the procedure will show less variation from high to low arousal when there is no affective interference (Hussain, Calvo & Chen, 2014). Therefore, it cannot be used to compare different variations of cognitive load during usability testing when there is not a significant difference between the mental loads. Although, there are different studies that have used facial expression in order to evaluate the usability of different interfaces, there are not many studies in the area of evaluating facial expression in measuring cognitive load for usability test purposes. See Table 18 for relevant studies that used facial expression alone or in conjunction with other measurement methods.

The reviewed studies demonstrate that facial-expression analysis, whether used independently or in combination with other cognitive load measures, provides an effective method for assessing usability and cognitive load. Facial-expression data were frequently analysed alongside physiological indicators such as EDA and HRV (Dargent et al., 2019; Thüring & Mahlke, 2007), performance outcomes (Machado et al., 2018; Thüring & Mahlke, 2007; Xu et al., 2018), self-reported usability or satisfaction questionnaires (Thüring & Mahlke, 2007; Xu et al., 2018), and eye-movement measures including pupil dilation or blink rate (Corcoran et al., 2012; Dargent et al., 2019). Across these investigations, increased cognitive load and reduced usability were consistently associated with negative or strained facial expressions (e.g., frowning and brow tension), whereas positive affective expressions corresponded to lower mental effort. The convergence of facial-expression patterns with behavioural, subjective, and physiological indicators provides applied evidence of the convergent validity of facial-expression analysis for detecting user workload and emotional engagement during usability evaluation, even though most studies were designed for applied assessment rather than formal psychometric validation.

## Analysis

In this section, we will explore the answers to the review questions based on the reviewed literature.

### Q1 What Are the Existing Cognitive Load Measurement Methods That Have Been Effectively Used in the Area of Software Usability?

The review revealed that almost all the cognitive load measurement methods including NASA-TLX, Usability questionnaire, EEG, fNIRS, Dual-task paradigm method, Performance measures (task completion and time duration), Mouse dynamics, Linguistic Features, Fixations, Saccades, Pupil dilation, Blink rate, EDA, HRV, Blood pressure, and Facial expressions can and have been used to evaluate software usability. The only methods that have not been used in any of the existing studies are MEG and fMRI.

### Q2 What Are the Most Common Cognitive Load Measurement Methods That Can Be Used in the Area of Software Usability?

**Figure 2.**
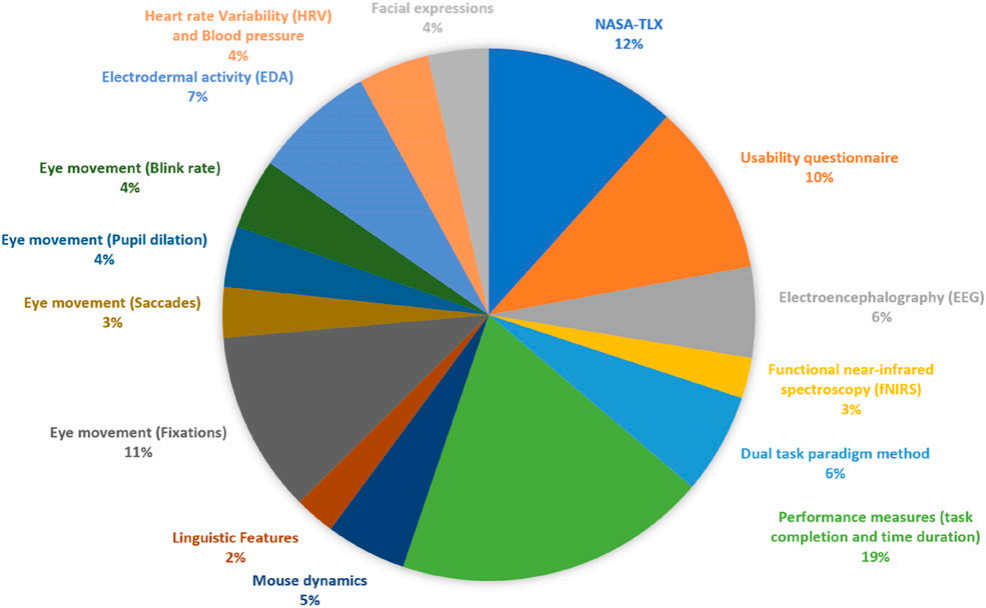
The percentage of each cognitive load measurement method that is used to evaluate usability.

Based on the current studies, performance measures (task completion and time duration) are the most used cognitive load measurement methods, appearing in 19% of the studies. This is followed by NASA-TLX (12%), eye movements (fixations) (11%), usability questionnaires (10%), EDA (7%), EEG (6%), and the dual-task paradigm method (6%), making them the most common usability evaluation approaches. Together, these methods account for 71% of the studies. Less commonly used methods include mouse dynamics (5%), HRV and blood pressure (4%), facial expressions (4%), eye movements (blink rate) (4%), pupil dilation (4%), saccades (3%), and fNIRS (3%). The least common method, linguistic features, appears in only 2% of the studies. Figure 2 illustrates the percentage of each evaluation method used in the reviewed studies.

### Q3 What Types of Software Are Evaluated by Using Cognitive Load Measurement Methods?

**Figure 3.**
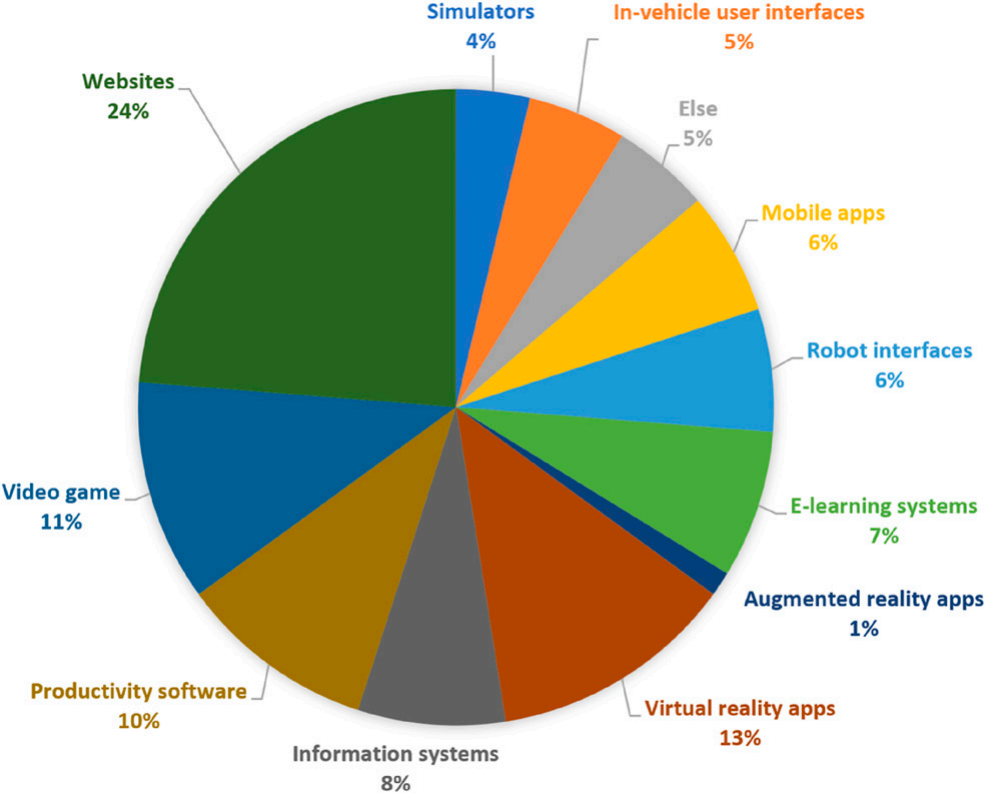
The percentage of each type of user interfaces that are evaluated using cognitive load measurement methods.

Different types of software have been evaluated using cognitive load measurement techniques. As shown in Figure 3, the most commonly evaluated software category is websites, accounting for 24% of the studies. Following this, virtual reality applications (13%), video games (11%), and productivity software (10%) are the next most frequently assessed categories. Information systems (8%) and e-learning systems (7%) are also widely studied. Meanwhile, mobile apps (6%), robot interfaces (6%), and in-vehicle user interfaces (5%) are evaluated less frequently. The least studied categories include simulators (4%), augmented reality (1%), and other miscellaneous software (5%).

### Q4 What Types of Software Have Been Evaluated by Using Each Cognitive Load Measurement Method?

**Figure 4.**
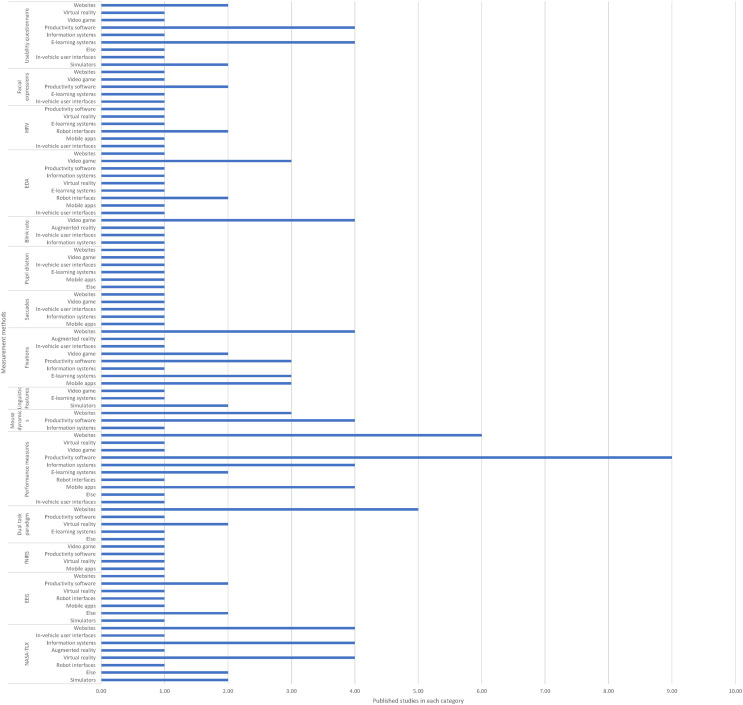
Different types of software that are evaluated using each cognitive load measurement method.

To answer this research question, different types of software were grouped based on the cognitive load measurement methods used to evaluate them. Our key findings indicate that performance measures (task completion and time duration) are the most widely used method across multiple software categories, particularly for websites, mobile apps, productivity software, information systems, and e-learning systems. Usability questionnaires are most commonly used to assess productivity software, websites, and e-learning systems, while NASA-TLX is frequently applied to evaluate websites, information systems, and virtual reality applications. EEG is primarily used to assess mobile apps, and productivity software, whereas the dual-task paradigm method is mostly used for evaluating websites and virtual reality. Fixations are commonly employed to assess websites, productivity software, e-learning systems, video games, and mobile apps, whereas blink rate is most frequently used to evaluate video games. Additionally, EDA is often utilised to assess video games, and robot interfaces, while HRV and blood pressure are commonly used for evaluating robot interfaces. Mouse dynamics are predominantly used to evaluate websites and productivity software, while linguistic features are primarily used in simulator evaluations. Finally, facial expressions are most frequently analysed in the evaluation of productivity software. For more details, see Figure 4.

### Q5 What Cognitive Load Measurement Methods Have Been Used for Evaluating the Usability of Each Type of Software?

**Figure 5.**
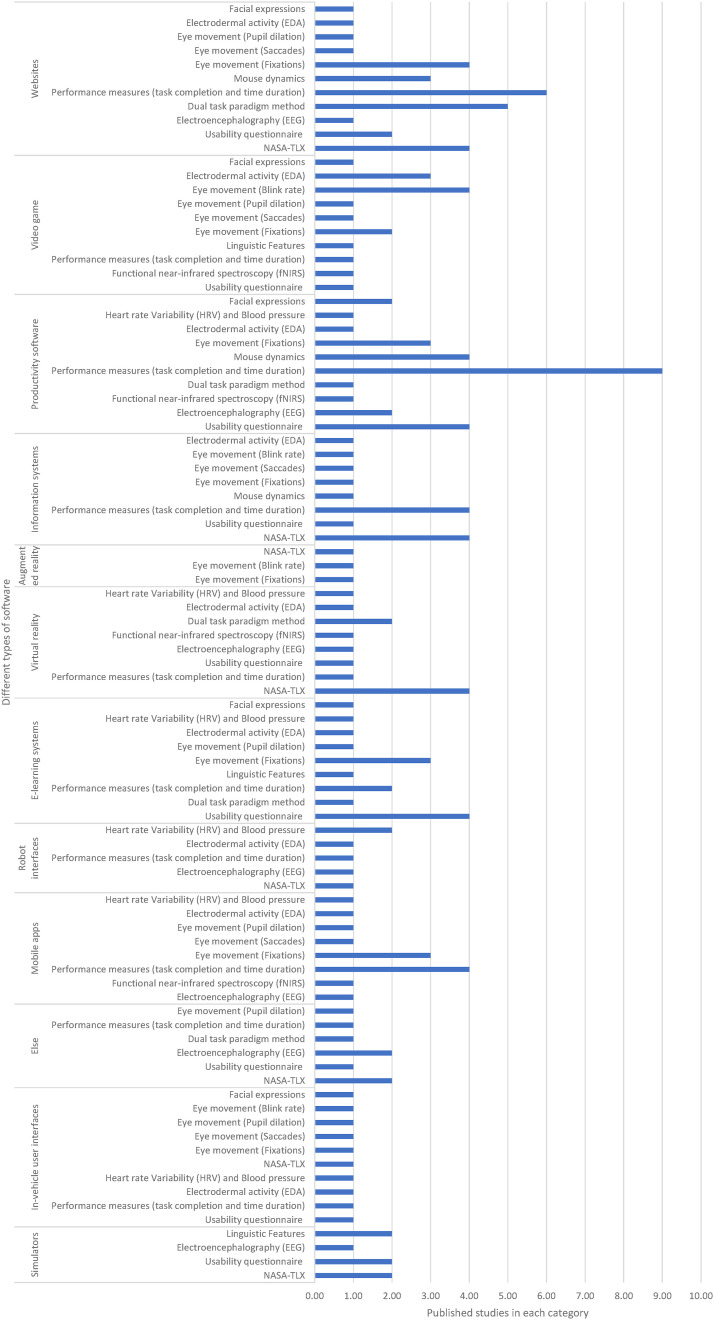
Cognitive load measurement methods that have been used for evaluating the usability of each type of software.

To answer this research question, different cognitive load measurement methods are grouped by software category. As key findings, we observed that websites are most often evaluated using NASA-TLX, the dual-task paradigm, performance measures, mouse dynamics, and fixations. Video games are primarily assessed using blink rate and electrodermal activity. Productivity software is commonly evaluated using usability questionnaires, performance measures, and mouse dynamics. Information systems are frequently assessed using NASA-TLX and performance measures. E-learning systems are most often evaluated using usability questionnaires and fixations, while mobile apps are primarily assessed using performance measures and fixations. Finally, virtual reality applications are typically evaluated using NASA-TLX. For more details, see Figure 5.

Although Figures 4 and 5 present the frequency of each cognitive load measurement method identified across studies, it should be noted that several categories include only a single publication. Therefore, these figures should be interpreted as showing coverage rather than strength of evidence. The visualisations depict the measurement methods most frequently employed by researchers in usability evaluation studies, but they do not necessarily reflect the relative reliability or validity of those methods. In cases where only one or two studies support a particular method, our recommendations are guided chiefly by the methodological rigour, and the degree of convergence with related findings from complementary measurement methods, rather than by frequency of use alone. To increase transparency, the strength of evidence for each measure is outlined in the Discussion section and summarised in Table 19 (see Appendix).

### Q6 How Valid Are the Identified Cognitive Load Measurement Methods, Particularly in Terms of Convergent Validity, for Evaluating Software Usability?

In addressing Q6, this review evaluated the validity of cognitive load measurement methods in usability testing by examining whether each technique behaved in accordance with theoretical expectations and showed convergence with complementary indicators. Because most studies did not report formal validity coefficients, validity was assessed through convergent validity, understood here as a key source of evidence contributing to overall construct validity (Campbell & Fiske, 1959; Cronbach & Meehl, 1955; Messick, 1995a, 1995b). A method was considered to provide convergent evidence when its results systematically followed expected patterns of interface complexity or corresponded with other measurements such as subjective ratings, behavioural performance, or physiological responses (Cook & Beckman, 2006). This approach allowed us to determine whether each method demonstrated the type of empirical behaviour that would support its validity in applied usability contexts.

Across studies, performance measures, dual-task paradigms, and physiological indicators such as HRV and EDA demonstrated strong convergent validity. These methods consistently revealed predictable associations, such as increased task time, error rates, or autonomic arousal under higher cognitive demands, providing robust evidence that they were sensitive to underlying fluctuations in cognitive load (Antonenko & Niederhauser, 2010; Bong et al., 2016; Brunken et al., 2003; Khawaja, Ruis, & Chen, 2007; Solovey et al., 2014; Yin et al., 2008). At the same time, physiological indicators were susceptible to extraneous influences such as stress, fatigue, or environmental variation, which occasionally reduced convergence in naturalistic settings.

Self-report methods such as NASA-TLX and post-task usability questionnaires provided consistent and interpretable workload ratings and exhibited convergent validity when their results aligned with behavioural or physiological indicators. Their validity was strongest in tasks with sustained cognitive demands, whereas rapid workload fluctuations or emotional interference sometimes weakened convergence with other measures due to reliance on retrospective judgement.

Neurophysiological methods, including EEG and fNIRS, showed convergent validity in controlled environments, where both modalities reliably detected neural patterns associated with increased task complexity (e.g., frontal theta power or oxygenation changes). However, variability arising from signal noise, sensor placement, and environmental factors limited convergence across sessions and participants, constraining their validity in applied usability contexts.

Eye-tracking measures, including fixation count, fixation duration, saccades, and pupil dilation, demonstrated robust convergent validity when calibration and lighting were stable (Chen et al., 2011; Jiang et al., 2018; Wang et al., 2020). Fixation-based metrics consistently increased with task difficulty and mirrored changes in performance indicators such as error rates and task-completion times. Convergence weakened when fatigue, emotional states, or lighting fluctuations confounded pupil-based indicators.

Behavioural measures such as mouse dynamics and linguistic features demonstrated emerging convergent validity. Several studies documented systematic associations between movement variability or speech characteristics and task difficulty (Darejeh, Marcus & Sweller, 2021; Kortum & Acemyan, 2016), but inconsistent replication across interface types, tasks, and user populations limited the strength of convergent evidence currently available for these measures.

Overall, the findings indicate that while no single method fully captures cognitive load across all usability scenarios, several methods, including NASA-TLX, performance measures, eye-tracking, EEG/fNIRS, and selected physiological indicators, exhibit the strongest convergent validity, providing consistent and theoretically aligned evidence across studies. From a construct perspective, methods that repeatedly showed strong convergent validity offer the most defensible evidence supporting their construct validity as cognitive load measures.

From an applied usability standpoint, validity is enhanced when multiple complementary methods are combined. Converging evidence from subjective, behavioural, and physiological indicators strengthens interpretability and mitigates limitations inherent to single-method approaches. Accordingly, integrating lightweight behavioural metrics (e.g., performance measures and mouse dynamics), subjective tools (e.g., NASA-TLX), and selected physiological signals (e.g., pupil dilation or EDA) provides a practical and valid strategy for capturing cognitive load in usability evaluations while balancing ecological feasibility, interpretability, and methodological rigour.

## Discussion

In this review, multiple CL measurement methods applicable to usability evaluations were analysed. This discussion outlines how these methods can be selected and applied through the lens of CLT, with a focus on their validity, interpretive usefulness, and practical integration into usability testing. In usability contexts, cognitive load arises from both intrinsic task complexity and extraneous design inefficiencies and their impact on a user’s mental effort, and most methods can capture aspects of both depending on task characteristics and interface design.

Subjective approaches such as Likert-scale questionnaires and NASA-TLX (Ayres, 2006; Paas, 1992; Paas et al., 2016) remain the most commonly used measures of perceived mental effort due to their simplicity, low cost, and adaptability across domains. Their limitations, retrospective self-report, recall bias, and sensitivity to perceived expectations, are well documented. However, these methods show strong convergent validity when subjective ratings align with behavioural or physiological indicators, as demonstrated by repeated convergence between NASA-TLX scores, performance metrics, and usability ratings (Brendle et al., 2020; Darejeh et al., 2024a, 2024b; Koć-Januchta et al., 2022; Richardson et al., 2019; Rockman et al., 2020).

Objective neurophysiological techniques such as EEG and fNIRS (Baldwin & Cisler, 2017; Van Mierlo et al., 2012) offer fine-grained temporal resolution and capture real-time fluctuations in mental effort. These methods exhibit convergent validity when neural patterns follow predicted CLT trends and align with subjective or performance-based measures (Baig & Kavakli, 2018; Cano et al., 2021; Plazak et al., 2019). Their primary limitations relate to intrusiveness, cost, and the need for technical expertise, making them more suitable for controlled than applied settings.

The dual-task paradigm (Schmutz et al., 2009; Van Nuland, 2017) provides a cost-effective way to estimate attentional demand by measuring decrements on a secondary task. Its validity emerges from consistent alignment with CLT’s assumptions about limited working-memory capacity. When dual-task results converge with subjective or behavioural indicators (Giraud et al., 2018; Na, 2021; Wenk et al., 2023), the method demonstrates clear convergent validity. However, secondary-task interference can limit ecological realism, requiring careful calibration across task types.

Performance metrics such as completion rate, error count, and task duration (Antonenko & Niederhauser, 2010; Brunken et al., 2003; Cranford et al., 2014; Holmqvist et al., 2011) offer highly interpretable indicators of cognitive load. Their validity becomes evident when performance patterns align with predicted changes in intrinsic or extraneous load. Although performance data alone cannot distinguish between task complexity and usability problems, these measures show strong convergent validity when integrated with subjective or physiological data (Da Costa et al., 2013; Darejeh et al., 2024a, 2024b; Holden et al., 2020; Huang, Eades & Hong, 2009; Rhodes et al., 2019).

Behavioural and linguistic measures, such as mouse-movement dynamics (Darejeh, Marcus & Sweller, 2021; Grimes & Valacich, 2015; Hibbeln et al., 2014; Kortum & Acemyan, 2016) and speech- or text-based linguistic features (Khawaja, Chen & Marcus, 2012, 2014; Oviatt, 2006; Tuček, Mount & Abbass, 2012), are non-intrusive and suitable for ecologically valid environments. Although their interpretive stability may be influenced by user habits or contextual variation (Chen et al., 2016), many studies report emerging convergent validity when behavioural or linguistic indicators align with subjective or performance-based measures.

Physiological measures, including eye-tracking, EDA, HRV, and facial-expression analysis, offer sensitive, objective indicators of mental effort. Eye-tracking (Bruneau et al., 2002; Calvo et al., 2022; Cheng & Wei, 2018; Huang & Zhang, 2022) excels at revealing visual-attention patterns, though lighting and fatigue can affect measurement quality. EDA and HRV (Barathi et al., 2020; Collins et al., 2019; Rajavenkatanarayanan et al., 2020; Solovey et al., 2014) provide continuous physiological markers of arousal and workload, while facial-expression analysis (Dargent et al., 2019; Thüring & Mahlke, 2007; Xu et al., 2018) captures affective states interacting with cognitive load. Across these modalities, convergent validity is frequently observed when physiological signals align with behavioural or self-report data (Gupta et al., 2019; Guerberof, Moorkens & Brien, 2021; Zardari, Hussain & Arain, 2021; Zhou et al., 2020).

Selecting the most suitable method requires considering the interface type, mobility constraints, and desired level of precision. For desktop systems, most cognitive load measurement methods are feasible due to stable posture and controlled environments. For mobile applications, where users move dynamically, subjective assessments, performance measures, and dual-task paradigms are generally more practical, though lightweight wearable eye-trackers may be appropriate. For immersive environments such as VR, robotics, or smart systems, less restrictive measures (e.g., performance metrics, dual-task paradigms, and linguistic analysis) tend to be more practical, while wireless sensors enable physiological monitoring in these contexts.

Combining methods yields a richer understanding of cognitive workload. Pairing subjective self-reports with objective measures such as eye-tracking or performance metrics can help reconcile perceived and observed effort. Integrating behavioural and physiological data clarifies how attention, arousal, and interface design shape cognitive demands. However, combinations that physically interfere with each other, such as mouse tracking with finger-mounted EDA sensors, should be avoided. The optimal combination depends on study goals, resource constraints, and ecological realism. A summary of advantages and limitations for each method appears in Table 19 in the Appendix.

**Figure 6.**
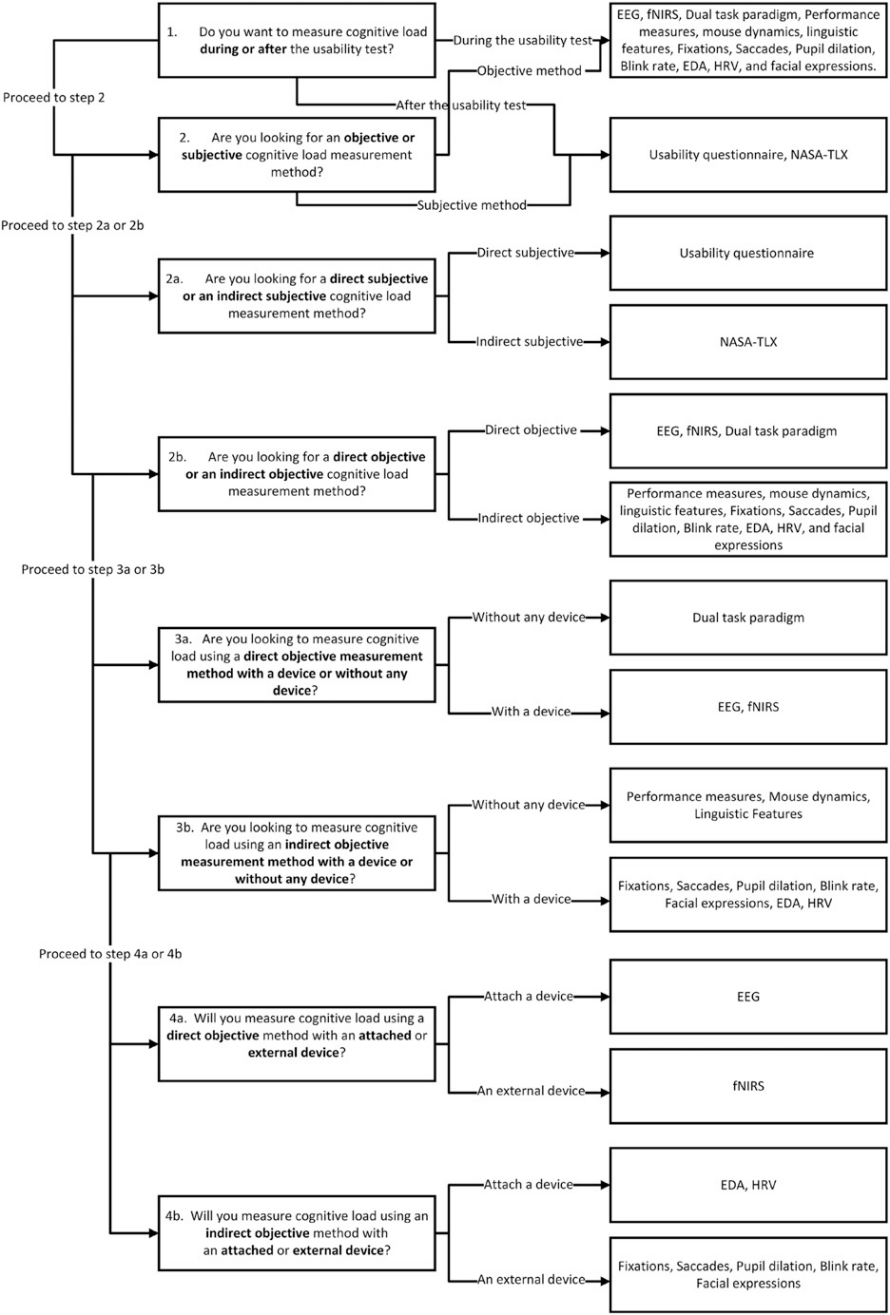
The framework for choosing an appropriate cognitive load measurement method in the context of usability.

Based on this review, we proposed a framework to guide usability testers in selecting the appropriate cognitive load measurement method for conducting accurate usability evaluations (Figure 6). This framework consists of numbered boxes that illustrate the flow of the decision process, helping researchers and practitioners narrow down available methods according to key methodological characteristics, such as whether cognitive load is measured during or after the usability test, whether a direct or indirect approach is preferred, and whether measurement involves device-based or non-device-based techniques.

Although these distinctions form part of the framework’s structure, they are not intended as theoretical claims about the nature of the measures, but rather as a *conceptual decision aid* for identifying an initial set of candidate approaches. Once this preliminary set is established, practitioners can refine their selection based on situational factors such as application type, target user population, testing environment, cost, and level of intrusiveness. In this way, the framework remains flexible and applicable across both research and applied usability contexts, supporting informed and context-sensitive decision making. This framework is supported by Brunken et al. (2003); Schmutz et al. (2009); and Kosch et al. (2023).

Based on this framework, we can measure the cognitive load of users during or after the usability test (box number 1 of the framework) using either objective or subjective (box number 2 of the framework) measurement methods. If the cognitive load is measured during the usability test using objective measurement methods (boxes number 1 plus 2 of the framework), we can employ methods including EEG, fNIRS, Dual-task paradigm, Performance measures, mouse dynamics, linguistic features, Fixations, Saccades, Pupil dilation, Blink rate, EDA, HRV, and facial expressions (The box that is next to the box #1). However, if the cognitive load is measured after the usability test using subjective measurement methods (box number 1 plus 2 of the framework), we can use the Usability questionnaire and NASA-TLX (The box that is next to the box #2). Direct subjective method is the Usability questionnaire and indirect subjective method is the NASA-TLX (box number 2a of the framework). Direct objective methods include EEG, fNIRS, and Dual-task paradigm, while indirect objective methods include Performance measures, mouse dynamics, linguistic features, Fixations, Saccades, Pupil dilation, Blink rate, EDA, HRV, and facial expressions (box number 2b of the framework).

When measuring cognitive load using direct objective measurement methods, we have two options: using a device or measuring without using any device. Without using any device, we can utilise the dual-task paradigm, and with the use of a device, we can choose between EEG or fNIRS (box number 3a of the framework). Furthermore, we can measure cognitive load using indirect objective measurement methods either with the use of a device or without using any device. When measuring without using any device, we can employ Performance measures, Mouse dynamics, and Linguistic Features. On the other hand, when using a device, we can utilise Fixations, Saccades, Pupil dilation, Blink rate, Facial expressions, EDA, and HRV (box number 3b of the framework). Finally, when using a direct objective measurement method with a device, we can use a device attached to users such as EEG, or an external device like fNIRS (box number 4a of the framework). Similarly, when using indirect objective measurement methods with a device, we can use a device attached to users like EDA and HRV, or external devices like Fixations, Saccades, Pupil dilation, Blink rate, and Facial expressions (box number 4b of the framework).

Here are two examples of how our framework can be used to evaluate the usability of different types of interfaces.

Example 1 (Evaluating a Mobile App Interface): In the context of evaluating a new mobile banking app, usability testers can implement the proposed framework to measure cognitive load during and after the usability test. During the test, objective methods such as EEG and fNIRS (direct objective) can be used to continuously monitor brain activity as participants navigate the app. Additionally, mouse dynamics and touch interactions (indirect objective) can be tracked to assess ease of navigation, while eye-tracking glasses can monitor pupil dilation and blink rate (indirect objective) to identify moments of increased cognitive load. Performance measures (indirect objective) such as task completion time and success rates for tasks like transferring money or checking account balances can also be recorded. After the usability test, subjective methods such as a usability questionnaire (direct subjective) and the NASA-TLX (indirect subjective) can be administered to gather participants’ feedback on their experience, ease of use, and perceived workload.

Example 2 (Evaluating an e-Learning Platform): When evaluating an online education platform designed for university students, usability testers can use the proposed framework to assess cognitive load during and after the usability test. During the test, objective methods such as a dual-task paradigm (direct objective) can be implemented, where a secondary task like tapping a button when a visual cue appears measures cognitive load while completing e-learning modules. Eye-tracking (indirect objective) can monitor fixations and saccades to identify areas causing confusion, while sensors for heart rate variability and electrodermal activity (indirect objective) can provide indicators of cognitive load during learning tasks. Performance measures (indirect objective) like time on task and error rates for activities such as quizzes or interactive simulations can also be tracked. After the test, subjective methods such as a usability questionnaire (direct subjective) and the NASA-TLX (indirect subjective) can be used to gather feedback on user experience, ease of use, and perceived workload.

## Limitations and Future Work

Although we tried to consider different criteria in our proposed framework, it has some limitations. First of all, it can be complex for those with limited experience in usability evaluation to implement effectively. Usability evaluators must navigate through detailed branching and multiple decision points, requiring a solid understanding of cognitive load theories and measurement techniques. Additionally, many of the recommended methods, such as EEG and fNIRS, necessitate specialised and often expensive equipment, which may not be accessible to all researchers or organisations.

Context-specific validity is another concern, as the framework may not account for the diverse environments and tasks in which cognitive load measurements are conducted. Different usability testing contexts can significantly influence the appropriateness and effectiveness of various cognitive load measurement methods. For example, methods suitable for a controlled laboratory setting might not be applicable or reliable in real-world or field environments. Furthermore, the complexity of various tasks and the specific attributes of different user populations can introduce variability that the framework might not fully address. Moreover, users need to be careful not to try to apply this usability evaluation framework too broadly to other HCI contexts where it may not be relevant.

Future research can utilise our proposed framework to assess its effectiveness and validity. Additionally, future studies should focus on the reliability and validation of the cognitive load measurement methods identified in this study to determine the most effective and reliable techniques for measuring usability. Investigating ways to minimise the intrusiveness of physiological measurement devices, such as EEG and heart rate monitors, while maintaining accuracy, can also be explored. In particular, future work could build on a comparative performance analysis of different types of EEG devices, such as scalp EEG and ear EEG-based P300 ambulatory brain–computer interfaces (Gupta et al., 2022), or different models of EMOTIV BCIs, using Riemannian geometry and deep learning-based approaches. This would evaluate the performance of different EEG configurations in measuring cognitive load. Moreover, many of the cognitive load measures identified in this review could be further evaluated for use in other broader contexts.

## Conclusions

This paper provides a comprehensive review and analysis of various cognitive load measurement methods for evaluating the usability of different types of user interfaces. By examining 87 articles, we have identified the most effective methods for measuring cognitive load during usability testing, highlighting their advantages and disadvantages. As a summary, a cognitive load measurement method that will be used during a usability test process should have two characteristics: (a) it should not create any discomfort for users and (b) it should measure the cognitive load continuously, not just at the end of the session.

Although methods such as brain activity measurement procedures including EEG, and physiology measures such as heart rate variability and electrodermal activity can measure cognitive load continuously and objectively, they can cause stress or distraction, especially when the experiment duration is long (Van Mierlo et al., 2012). Furthermore, the information from eye-tracking methods can be affected by personal or environmental factors such as tiredness or brightness (Siegle, Steinhauer, & Thase, 2004). Hence, proper consideration has to be made when using such methods.

By considering the advantages and disadvantages of different measurement methods, it can be concluded that the most suitable methods to measure cognitive load during a usability test are performance on task, time on task, dual-task paradigms especially the tapping method, mouse dynamics, facial expressions, and linguistic features if applicable. These methods measure the mental load continuously without causing any discomfort or distraction for the participants. They also do not need expensive equipment and can be used by non-expert people as well. In addition to the above methods, since self-reported difficulty or mental effort questionnaires can provide valuable information and do not create any discomfort for the participants, they can be used as a complementary method at the completion of a usability test. Finally, in order to conduct an accurate usability test, a combination of subjective and objective cognitive load measurement methods should be utilised to compensate for the shortcomings of each method and increase the reliability of the results.

Finally, our proposed framework serves as a valuable guide for usability experts, cognitive load researchers, and those involved in evaluating the usability of interfaces across various domains, including information systems, e-learning software, video games, and immersive technologies like virtual and augmented reality. This framework aids in selecting the most appropriate cognitive load measurement methods tailored to specific contexts, ensuring more accurate and reliable usability evaluations.

## Key Points

• This systematic review analyses 87 studies to identify cognitive load measurement methods applied in software usability testing.
• It categorises cognitive load measurement methods into subjective and objective types and evaluates their applicability, strengths, and limitations across various interface types.
• The most commonly used methods include performance measures, NASA-TLX, and eye-tracking-based fixation analysis.
• A practical framework is proposed to guide researchers and practitioners in selecting appropriate cognitive load measurement techniques tailored to specific interface evaluation goals.

## Appendix

**Table 19.** Advantages and Disadvantages of Each Measurement’s Method.

When and Why to Use Each Usability Measurement Method	Advantages	Disadvantages
NASA-TLX is used to assess perceived workload in usability evaluations, suitable for comparing designs, testing in real-world conditions, and critical environments like healthcare and simulations to optimise user experience and safety by managing cognitive load effectively.	1. Multi-dimensional: Captures various aspects of user experience across six dimensions.	1. Subjectivity: Variability due to different user interpretations and influences.
	2. Subjective assessment: Provides insights based on users’ personal experiences.	2. Potential bias: Ratings can be influenced by overall or recent experiences.
	3. Widely used and accepted: Well-validated and credible within the scientific community.	3. Time-consuming: Pairwise comparison step can be burdensome in large studies.
	4. Ease of use: Simple and quick for users to complete.	4. Not task-specific: May not capture all specific usability issues and might need additional measures.
	5. Versatility: Applicable to a wide range of tasks and contexts.	
EEG is beneficial in usability evaluation for real-time assessment of cognitive processes and emotional responses, ideal for interactive systems, video games, and research to optimise user experience and interface design based on neurological feedback.	1. Objective measurement: Provides unbiased data on brain activity.	1. Complexity: Requires specialised knowledge to analyse and interpret.
	2. Real-time data: Captures moment-by-moment changes in cognitive load and emotions.	2. Setup and equipment: Time-consuming and expensive to set up.
	3. Detailed insights: Identifies specific cognitive and emotional states.	3. Environmental sensitivity: Prone to noise and movement artifacts affecting data quality.
	4. Rich data: Uncovers subtle usability problems not evident through other methods.	4. User discomfort: Wearing the EEG cap can be uncomfortable or distracting.
		5. Data interpretation: Challenging to link specific brain wave patterns to usability issues; often needs supplementary data.
fNIRS is suited for usability evaluation in applications requiring non-invasive monitoring of brain activity, such as interactive systems, rehabilitation devices, and cognitive workload assessments in diverse user environments. It provides insights into cognitive engagement and task performance, guiding improvements in user interface design and usability.	1. Good temporal resolution: Captures changes in brain activity over time.	1. Lower spatial resolution: Less precise in pinpointing exact locations of brain activity.
	2. Less sensitive to movement: Suitable for tasks with head or physical movement.	2. Complex data analysis: Requires specialised knowledge to analyse and interpret.
	3. Measures hemodynamic responses: Provides insights into mental workload and cognitive states.	3. Cost and equipment: Expensive and requires specialised equipment and expertise.
		4. Susceptible to external factors: Hair thickness and skin tone can affect signal quality.
Dual-task paradigm method is effective in usability evaluation for assessing multitasking capabilities and cognitive workload in complex interactive systems, simulations, and tasks requiring simultaneous attention to multiple stimuli or actions.	1. Sensitive to usability issues: Reveals hidden usability problems.	1. Increased complexity: Complex to design and interpret.
	2. Real-world relevance: Simulates real-world multitasking scenarios.	2. Participant fatigue: Can lead to exhaustion, affecting data quality.
	3. Objective data: Provides quantitative performance metrics.	3. Interference between tasks: Secondary task might skew results.
	4. Versatility: Adaptable to various task types and contexts.	4. Not always feasible: Unsuitable for some usability test task combinations.
Performance measures are valuable in usability evaluation for assessing efficiency and effectiveness in interactive systems, software applications, and digital interfaces. They provide quantitative insights into user proficiency, task complexity, and overall usability improvements.	1. Objective data: Quantifiable and easy to analyse.	1. Limited insight into user experience: Misses qualitative aspects like satisfaction.
	2. Easy to implement: Simple measurement and recording.	2. Potential for superficial results: May overlook deeper issues.
	3. Direct indicators of usability: Reflects efficiency and effectiveness.	
	4. Broad applicability: Usable across various systems and tasks.	
	5. Baseline comparison: Facilitates comparison with baseline data or different versions of the same application.	
Mouse dynamics are useful in usability evaluation for assessing user behaviour, engagement, and task performance in software applications, websites, and interactive interfaces. They provide insights into user interactions, navigation patterns, and usability issues related to interface design and user experience optimisation.	1. Objective data: Quantifiable metrics on user interactions.	1. Limited insight into user intentions: Misses cognitive processes and intentions.
	2. Real-Time tracking: Captures real-time user behaviour.	2. Potential for misinterpretation: Difficult to interpret without context.
	3. Fine-Grained analysis: Detailed insights into navigation and interaction.	3. Environmental factors: Influenced by user dexterity and hardware differences.
	4. Non-intrusive: Does not disrupt natural interaction.	4. Incomplete usability picture: Needs supplementary measures for a comprehensive view.
Linguistic features are valuable in usability evaluation for analysing user feedback, sentiments, and interactions in text-based interfaces, chatbots, and customer support systems. They provide insights into user satisfaction, comprehension, and usability issues related to language clarity and effectiveness in communication.	1. Rich qualitative data: In-depth insights into user experiences.	1. Subjectivity: Interpretation can be influenced by the analyst’s perspective.
	2. Contextual understanding: Understands the context behind user actions.	2. Time-consuming: Labour-intensive data collection and analysis.
	3. User-centred: Directly captures user perspectives.	3. Requires expertise: Needs language and qualitative analysis expertise.
	4. Identifies specific issues: Pinpoints usability problems and frustrations.	4. Potential for inconsistent data: Variable data quality due to unclear user articulation.
	5. Adaptable to various methods: Complements other usability methods.	5. Limited quantitative data: Needs supplementary quantitative measures.
Eye movements (fixations and saccades) are beneficial in usability evaluation for studying visual attention, information processing, and interface design effectiveness in applications such as websites, advertisements, and complex data displays. They provide insights into user navigation patterns, attention allocation, and usability issues related to visual layout and information hierarchy.	1. Direct measurement of attention: Directly observes what users visually attend to.	1. Influence of task constraints: Behaviour may be influenced by task instructions.
	2. Objective data: Quantitative metrics on eye movements.	2. Equipment and setup: Requires specialised and costly equipment.
	3. Real-time analysis: Observes and analyses behaviour in real time.	3. Interpretation challenges: Needs expertise for accurate data interpretation.
	4. Visual attention mapping: Maps user attention patterns.	
	5. Temporal dynamics: Studies the speed and efficiency of visual search.	
	6. Non-invasive: Does not require physical contact.	
Pupil dilation and blink rate are useful in usability evaluation for assessing cognitive load, emotional responses, and user engagement in applications involving high cognitive demand, visual stimuli, and interactive tasks. They provide real-time indicators of user attention, arousal levels, and usability challenges related to interface complexity and user experience design.	1. Very sensitive to cognitive load: Reflects mental effort.	1. Complex interpretation: Requires expertise for accurate analysis.
	2. Real-Time measurement: Tracks responses during interaction.	2. Individual variability: Responses vary between users.
	3. Non-invasive: Doesn’t require physical contact.	3. Environmental factors: Lighting and screen affect results.
	4. Objective data: Quantifies arousal and cognitive processing.	4. Equipment sensitivity: Requires precise equipment.
	5. Versatility: Applicable to various tasks.	5. Limited emotion differentiation: Doesn’t distinguish emotions well.
		6. Not universally applicable: Varies with age and health.
EDA is beneficial in usability evaluation for assessing emotional arousal, stress levels, and user engagement in applications such as gaming, VR, and interactive simulations. It provides insights into user responses to stimuli, interface design effectiveness, and usability improvements based on emotional and physiological feedback.	1. Sensitive to emotional responses: Measures arousal and stress levels.	1. Complex interpretation: Requires expertise in data analysis.
	2. Objective measurement: Quantifies physiological responses.	2. Equipment sensitivity: Needs specialised setup.
	3. Real-time monitoring: Tracks reactions during interaction.	3. Limited specificity: Doesn’t differentiate specific emotions.
	4. Complementary to other measures: Provides comprehensive insights.	4. User comfort: Potential discomfort with sensors which can affect usability test results.
HRV and blood pressure are valuable in usability evaluation for assessing physiological stress, cognitive workload, and user engagement in applications involving high-stress environments, medical devices, and safety-critical systems. They provide objective measures of user response, aiding in optimising interface design and usability to enhance user experience and safety.	1. Direct indicator of stress response: Indicate acute stress responses during usability tasks.	1. Equipment sensitivity: Requires precise equipment and setup.
	2. Quantitative measurement: Offering an objective assessment.	2. Environmental influence: Blood pressure can be influenced by factors such as ambient temperature, noise levels, and participant anxiety.
	3. Real-time monitoring: Real-time monitoring of cardiovascular changes during interaction.	3. Discomfort for participants: Not suitable for all usability studies.
	4. Clinical relevance: Can provide insights into overall physiological stress.	
	5. Complementary to other measures: Can provide a holistic view of user experience.	
Facial expressions are useful in usability evaluation for analysing user emotions, engagement, and feedback in applications such as video conferencing, virtual agents, and interactive media. They provide insights into user satisfaction, emotional responses to usability improvements focused on enhancing user experience through emotional engagement.	1. Non-invasive: Does not require physical contact.	1. Subjectivity in interpretation: Interpretation influenced by perception.
	2. Real-time observation: Provides immediate insights.	2. Limited specificity: May not differentiate subtle emotions.
	3. Rich qualitative data: Offers qualitative emotional information.	3. Environmental factors: Lighting and angles affect accuracy.
		4. Complex analysis: Requires expertise and resources.

## References

[bibr1-00187208261427867] Ahmed A. A. E. Traore I. (2007). A new biometric technology based on mouse dynamics. IEEE Transactions on Dependable and Secure Computing, 4(3), 165–179. 10.1109/tdsc.2007.70207

[bibr2-00187208261427867] Albers M. J. (2011, October). Tapping as a measure of cognitive load and website usability. In Proceedings of the 29th ACM international conference on design of communication (pp. 25–32). Association for Computing Machinery.

[bibr3-00187208261427867] Al-Shehri S. Gitsaki C. (2010). Online reading: A preliminary study of the impact of integrated and split-attention formats on L2 students’ cognitive load. ReCALL, 22(3), 356–375. 10.1017/s0958344010000212

[bibr4-00187208261427867] Anderson E. W. Potter K. C. Matzen L. E. Shepherd J. F. Preston G. A. Silva C. T. (2011). A user study of visualization effectiveness using EEG and cognitive load. Computer Graphics Forum, 30(3), 791–800. 10.1111/j.1467-8659.2011.01928.x

[bibr5-00187208261427867] Antonenko P. D. Niederhauser D. S. (2010). The influence of leads on cognitive load and learning in a hypertext environment. Computers in Human Behavior, 26(2), 140–150. 10.1016/j.chb.2009.10.014

[bibr6-00187208261427867] Astleitner H. Leutner D. (1996). Applying standard network analysis to hypermedia systems: Implications for learning. Journal of Educational Computing Research, 14(3), 285–303. 10.2190/W2GB-05NTVJRN-PGY9

[bibr7-00187208261427867] Ayres P. (2006). Using subjective measures to detect variations of intrinsic cognitive load within problems. Learning and Instruction, 16(5), 389–400. 10.1016/j.learninstruc.2006.09.001

[bibr8-00187208261427867] Ayres P. Lee J. Y. Paas F. van Merriënboer J. J. (2021). The validity of physiological measures to identify differences in intrinsic cognitive load. Frontiers in Psychology, 12, 702538. 10.3389/fpsyg.2021.702538 34566780 PMC8461231

[bibr9-00187208261427867] Baig M. Z. Kavakli M. (2018, December). Analyzing novice and expert user’s cognitive load in using a multi-modal interface system. In 2018 26th International Conference on Systems Engineering (ICSEng) (pp. 1–7). IEEE.

[bibr10-00187208261427867] Baldwin C. L. Cisler D. S. (2017). Neuroergonomic methods of assessing learning. In Cognitive load measurement and application (pp. 240–262). Routledge.

[bibr11-00187208261427867] Bandura A. Walters R. H. (1977). Social learning theory (Vol. 1, pp. 141–154). Prentice-Hall.

[bibr12-00187208261427867] Barathi S. C. Proulx M. O’Neill E. Lutteroth C. (2020, April). Affect recognition using psychophysiological correlates in high intensity VR exergaming. In Proceedings of the 2020 CHI conference on human factors in computing systems (pp. 1–15). Association for Computing Machinery.

[bibr13-00187208261427867] Barrios V. M. G. Gütl C. Preis A. M. Andrews K. Pivec M. Mödritscher F. Trummer C. (2004). AdELE: A framework for adaptive e-learning through eye tracking. In Proceedings of IKNOW (pp. 609–616). Graz.

[bibr311-00187208261427867] Beatty J. (1982). Task-evoked pupillary responses, processing load, and the structure of processing resources. Psychological Bulletin, 91(2), 276–292. 10.1037/0033-2909.91.2.276 7071262

[bibr14-00187208261427867] Bevan N. Carter J. Harker S. (2015, August). ISO 9241-11 revised: What have we learnt about usability since 1998? In International conference on human-computer interaction (pp. 143–151). Springer.

[bibr15-00187208261427867] Bong C. L. Fraser K. Oriot D. (2016). Cognitive load and stress in simulation. In Comprehensive healthcare simulation: Pediatrics (pp. 3–17). Springer.

[bibr16-00187208261427867] Boucsein W. (2012). Electrodermal activity. Springer Science & Business Media.

[bibr17-00187208261427867] Brendle C. Schütz L. Esteban J. Krieg S. M. Eck U. Navab N. (2020, October). Can a hand-held navigation device reduce cognitive load? A user-centered approach evaluated by 18 surgeons. In International conference on medical image computing and computer-assisted intervention (pp. 399–408). Springer.

[bibr18-00187208261427867] Brenner M. Shipp T. Doherty E. Morrissey P. (1985). Voice measures of psychological stress: Laboratory and field data. In Titze I. Scherer R. (Eds.), Vocal fold physiology, biomechanics, acoustics, and phonatory control (pp. 239–248). Denver Center for the Performing Arts.

[bibr19-00187208261427867] Brereton P. Kitchenham B. A. Budgen D. Turner M. Khalil M. (2007). Lessons from applying the systematic literature review process within the software engineering domain. Journal of Systems and Software, 80(4), 571–583. 10.1016/j.jss.2006.07.009

[bibr20-00187208261427867] Bruneau D. Sasse M. A. McCarthy J. (2002, April). The eyes never lie: The use of eye tracking data in HCI research. In Proceedings of the CHI 2002: Conference on Human Factors in Computing Systems (pp. 748–749). ACM.

[bibr21-00187208261427867] Brunken R. Plass J. L. Leutner D. (2003). Direct measurement of cognitive load in multimedia learning. Educational Psychologist, 38(1), 53–61. 10.1207/s15326985ep3801_7

[bibr23-00187208261427867] Bruser C. Stadlthanner K. de Waele S. Leonhardt S. (2011). Adaptive beat-to-beat heart rate estimation in ballistocardiograms. IEEE Transactions on Information Technology in Biomedicine, 15(5), 778–786. 10.1109/TITB.2011.2128337 21421447

[bibr24-00187208261427867] Brüser C. Winter S. Leonhardt S. (2012). Unsupervised heart rate variability estimation from ballistocardiograms. In Proceedings of the 7th international workshop on biosignal interpretation (Vol. 15, pp. 1–6). International Society for Bioelectromagnetism.

[bibr25-00187208261427867] Buccelletti E. Gilardi E. Scaini E. Galiuto L. Persiani R. Biondi A. Basile F. Silveri N. G. (2009). Heart rate variability and myocardial infarction: Systematic literature review and metanalysis. European Review for Medical and Pharmacological Sciences, 13(4), 299–307. https://www.europeanreview.org/article/650. 19694345

[bibr26-00187208261427867] Bus A. G. Takacs Z. K. Kegel C. A. (2015). Affordances and limitations of electronic storybooks for young children’s emergent literacy. Developmental Review, 35, 79–97. 10.1016/j.dr.2014.12.004

[bibr27-00187208261427867] Cacioppo J. T. Petty R. E. Losch M. E. Kim H. S. (1986). Electromyographic activity over facial muscle regions can differentiate the valence and intensity of affective reactions. Journal of Personality and Social Psychology, 50(2), 260–268. 10.1037//0022-3514.50.2.260 3701577

[bibr301-00187208261427867] Cain B. (2007). A review of the mental workload literature. (Contract No. RTO-TR-HFM-121-Part-II). Defence Research and Development Canada, Toronto Human System Integration Section, Toronto.

[bibr28-00187208261427867] Caldiroli C. L. Gasparini F. Corchs S. Mangiatordi A. Garbo R. Antonietti A. Mantovani F. (2023). Comparing online cognitive load on mobile versus PC-based devices. Personal and Ubiquitous Computing, 27(2), 495–505. 10.1007/s00779-022-01707-8 36594048 PMC9795953

[bibr29-00187208261427867] Calvo L. Christel I. Terrado M. Cucchietti F. Pérez-Montoro M. (2022). Users’ cognitive load: A key aspect to successfully communicate visual climate information. Bulletin of the American Meteorological Society, 103(1), E1–E16. 10.1175/bams-d-20-0166.1

[bibr300-00187208261427867] Campbell D. T. Fiske D. W. (1959). Convergent and discriminant validation by the multitrait-multimethod matrix, Psychological Bulletin 56(2), 81–105. 10.1037/h0046016 13634291

[bibr30-00187208261427867] Cano S. P. Soto J. Acosta L. Peñeñory V. Moreira F. (2021). Electroencephalography as an alternative for evaluating user eXperience in interactive systems. In WorldCIST (Vol. 1, pp. 435–444). Springer.

[bibr32-00187208261427867] Carlson N. (2013). Physiology of behavior. Pearson Education, Inc.

[bibr33-00187208261427867] Chan S. W. Sapkota S. Mathews R. Zhang H. Nanayakkara S. (2020). Prompto: Investigating receptivity to prompts based on cognitive load from memory training conversational agent. Proceedings of the ACM on Interactive, Mobile, Wearable and Ubiquitous Technologies, 4(4), 1–23. 10.1145/3432190.35846237

[bibr34-00187208261427867] Chandler P. Sweller J. (1991). Cognitive load theory and the format of instruction. Cognition and Instruction, 8(4), 293–332. 10.1207/s1532690xci0804_2

[bibr36-00187208261427867] Chen F. Zhou J. Wang Y. Yu K. Arshad S. Z. Khawaji A. Conway D. (2016). Robust multimodal cognitive load measurement. Springer.

[bibr37-00187208261427867] Chen O. Kalyuga S. Sweller J. (2015). The worked example effect, the generation effect, and element interactivity. Journal of Educational Psychology, 107(3), 689–704. 10.1037/edu0000018

[bibr38-00187208261427867] Chen S. Epps J. Ruiz N. Chen F. (2011, February). Eye activity as a measure of human mental effort in HCI. In Proceedings of the 16th international conference on intelligent user interfaces (pp. 315–318). Association for Computing Machinery.

[bibr39-00187208261427867] Cheng S. Wei Q. (2018, November). Design preferred aesthetic user interface with eye movement and electroencephalography data. In Proceedings of the 2018 ACM companion international conference on interactive surfaces and spaces (pp. 39–45). Association for Computing Machinery.

[bibr40-00187208261427867] Chynał P. Szymański J. M. Sobecki J. (2012, March). Using eyetracking in a mobile applications usability testing. In Asian conference on intelligent information and database systems (pp. 178–186). Springer.

[bibr41-00187208261427867] Clarke M. A. Schuetzler R. M. Windle J. R. Pachunka E. Fruhling A. (2020). Usability and cognitive load in the design of a personal health record. Health Policy and Technology, 9(2), 218–224. 10.1016/j.hlpt.2019.10.002

[bibr42-00187208261427867] Collins J. Regenbrecht H. Langlotz T. Can Y. S. Ersoy C. Butson R. (2019, October). Measuring cognitive load and insight: A methodology exemplified in a virtual reality learning context. In 2019 IEEE International Symposium on Mixed and Augmented Reality (ISMAR) (pp. 351–362). IEEE.

[bibr302-00187208261427867] Cook D. A. Beckman T. J. (2006). Current concepts in validity and reliability for psychometric instruments: Theory and application. The American Journal of Medicine, 119(2), 166.e7–166.e16. 10.1016/j.amjmed.2005.10.036 16443422

[bibr43-00187208261427867] Corcoran P. M. Nanu F. Petrescu S. Bigioi P. (2012). Real-time eye gaze tracking for gaming design and consumer electronics systems. IEEE Transactions on Consumer Electronics, 58(2), 347–355. 10.1109/tce.2012.6227433

[bibr44-00187208261427867] Cranford K. N. Tiettmeyer J. M. Chuprinko B. C. Jordan S. Grove N. P. (2014). Measuring load on working memory: The use of heart rate as a means of measuring chemistry students’ cognitive load. Journal of Chemical Education, 91(5), 641–647. 10.1021/ed400576n

[bibr45-00187208261427867] Critchley H. D. (2002). Electrodermal responses: What happens in the brain. The Neuroscientist, 8(2), 132–142. 10.1177/107385840200800209 11954558

[bibr303-00187208261427867] Cronbach L. J. Meehl P. E. (1955). Construct validity in psychological tests. Psychological Bulletin, 52(4), 281–302. 10.1037/h0040957 13245896

[bibr46-00187208261427867] Da Costa F. F. Schmoelz C. P. Davies V. F. Di Pietro P. F. Kupek E. de Assis M. A. A. (2013). Assessment of diet and physical activity of Brazilian schoolchildren: Usability testing of a web-based questionnaire. JMIR Research Protocols, 2(2), Article e31. 10.2196/resprot.2646 23958804 PMC3758065

[bibr47-00187208261427867] Darejeh A. Huynh L. Ji X. Marcus N. Polly P. (2024a). Assessing the efficacy of virtual reality-based laboratories versus real-life settings for PCR procedures, within medical science laboratories. In 2024 IEEE international symposium on mixed and augmented reality adjunct (ISMAR-Adjunct) (pp. 489–492). IEEE.

[bibr48-00187208261427867] Darejeh A. Marcus N. Sweller J. (2021). The effect of narrative-based E-learning systems on novice users’ cognitive load while learning software applications. Educational Technology Research & Development, 69(5), 1–23. 10.1007/s11423-021-10024-533551626

[bibr49-00187208261427867] Darejeh A. Marcus N. Sweller J. (2022). Increasing learner interactions with E-learning systems can either decrease or increase cognitive load depending on the nature of the interaction. L’Année Psychologique, 122(3), 405–437. 10.3917/anpsy1.223.0405

[bibr50-00187208261427867] Darejeh A. Mondal M. Marcus N. Vassar A. (2024b). Exploring the cognitive load effects of diverse virtual reality interaction methods in interactive educational platforms. In 2024 IEEE International Symposium on Mixed and Augmented Reality Adjunct (ISMAR-Adjunct) (pp. 435–438). IEEE.

[bibr51-00187208261427867] Dargent T. Karran A. Léger P. M. Coursaris C. K. Sénécal S. (2019). The influence of task types on user experience after a web interface update. Proceedings of the Eighteenth Annual Pre-ICIS Workshop on HCI Research in MIS. AIS Electronic Library.

[bibr52-00187208261427867] DeLeeuw K. E. Mayer R. E. (2008). A comparison of three measures of cognitive load: Evidence for separable measures of intrinsic, extraneous, and germane load. Journal of Educational Psychology, 100(1), 223–234. 10.1037/0022-0663.100.1.223

[bibr53-00187208261427867] Derick L. R. Gabriel G. S. Máximo L. S. Olivia F. D. Noé C. S. Juan O. R. (2020, November). Study of the user’s eye tracking to analyze the blinking behavior while playing a video game to identify cognitive load levels. In 2020 IEEE International Autumn Meeting on Power, Electronics and Computing (ROPEC) (Vol. 4, pp. 1–5). IEEE.

[bibr54-00187208261427867] Dobhan A. Wüllerich T. Röhner D. (2022). Eye-tracking and usability in (mobile) ERP systems. In International conference on enterprise information systems (pp. 403–423). Springer.

[bibr55-00187208261427867] Duran R. Zavgorodniaia A. Sorva J. (2022). Cognitive load theory in computing education research: A review. ACM Transactions on Computing Education (TOCE), 22(4), 1–27. 10.1145/3483843

[bibr56-00187208261427867] Ehmke C. Wilson S. (2007). Identifying web usability problems from eye-tracking data. In British computer society (p. 119–128). BCS Learning & Development Ltd.

[bibr57-00187208261427867] El Haddioui I. (2019). Eye tracking applications for E-Learning purposes: An overview and perspectives. In Cognitive computing in technology-enhanced learning (pp. 151–174). IGI Global.

[bibr58-00187208261427867] Engström J. Johansson E. Östlund J. (2005). Effects of visual and cognitive load in real and simulated motorway driving. Transportation Research Part F: Traffic Psychology and Behaviour, 8(2), 97–120. 10.1016/j.trf.2005.04.012

[bibr59-00187208261427867] Ferrari M. Quaresima V. (2012). A brief review on the history of human functional near-infrared spectroscopy (fNIRS) development and fields of application. NeuroImage, 63(2), 921–935. 10.1016/j.neuroimage.2012.03.049 22510258

[bibr60-00187208261427867] Fink M. C. Eisenlauer V. Ertl B. (2023). What variables are connected with system usability and satisfaction? Results from an educational virtual reality field trip. Computers & Education: X Reality, 3, 100043. 10.1016/j.cexr.2023.100043

[bibr61-00187208261427867] Forte G. Favieri F. Casagrande M. (2019). Heart rate variability and cognitive function: A systematic review. Frontiers in Neuroscience, 13, 710. 10.3389/fnins.2019.00710 31354419 PMC6637318

[bibr62-00187208261427867] Fowler A. Nesbitt K. Canossa A. (2019). Identifying cognitive load in a computer game: An exploratory study of young children. In 2019 IEEE Conference on Games (CoG) (pp. 1–6). IEEE. 10.1109/CIG.2019.8848064

[bibr63-00187208261427867] Frazier S. Pitts B. J. McComb S. (2022). Measuring cognitive workload in automated knowledge work environments: A systematic literature review. Cognition, Technology & Work, 24(4), 557–587. 10.1007/s10111-022-00708-0

[bibr64-00187208261427867] Freitas-Magalhães A. (2013). Facial expression of emotion: From theory to application. Leya.

[bibr65-00187208261427867] Fuller T. E. Garabedian P. M. Lemonias D. P. Joyce E. Schnipper J. L. Harry E. M. Benneyan J. C. Dalal A. K. (2020). Assessing the cognitive and work load of an inpatient safety dashboard in the context of opioid management. Applied Ergonomics, 85, 103047. 10.1016/j.apergo.2020.103047 32174343

[bibr66-00187208261427867] Galais T. Delmas A. Alonso R. (2019, December). Natural interaction in virtual reality: Impact on the cognitive load. In Proceedings of the 31st conference on l’Interaction homme-machine: Adjunct (pp. 1–9). Association for Computing Machinery.

[bibr67-00187208261427867] Gamboa H. Fred A. (2004, August). A behavioral biometric system based on human-computer interaction. In Biometric technology for human identification (Vol. 5404, pp. 381–392). International Society for Optics and Photonics.

[bibr68-00187208261427867] Gao Q. Sun Q. (2015). Examining the usability of touch screen gestures for older and younger adults. Human Factors, 57(5), 835–863. 10.1177/0018720815581293 25957042

[bibr69-00187208261427867] Geary D. (2008). An evolutionarily informed education science. Educational Psychologist, 43(4), 179–195. 10.1080/00461520802392133

[bibr70-00187208261427867] Geary D. (2012). Evolutionary educational psychology. In Harris K. Graham S. Urdan T. (Eds.), APA educational psychology handbook (Vol. 1, pp. 597–621). American Psychological Association. 10.1037/13273-020

[bibr71-00187208261427867] Geary D. Berch D. (2016). Evolution and children’s cognitive and academic development. In Geary D. Berch D. (Eds.), Evolutionary perspectives on child development and education (pp. 217–249). Springer.

[bibr72-00187208261427867] Georges V. Courtemanche F. Sénécal S. Léger P. M. Nacke L. Pourchon R. (2017, July). The adoption of physiological measures as an evaluation tool in UX. In International conference on HCI in business, government, and organizations (pp. 90–98). Springer.

[bibr73-00187208261427867] Gerjets P. W. Hesse F. W. H. Scheiter K. Eysink T. H. S. Opfermann M. (2009). Learning with hypermedia: The influence of representational formats and different levels of learner control on performance and learning behavior. Computers in Human Behavior, 25(2), 360–370. 10.1016/j.chb.2008.12.015

[bibr74-00187208261427867] Gevins A. Smith M. E. (2003). Neurophysiological measures of cognitive workload during human-computer interaction. Theoretical Issues in Ergonomics Science, 4(1-2), 113–131. 10.1080/14639220210159717

[bibr75-00187208261427867] Giraud S. Thérouanne P. Steiner D. D. (2018). Web accessibility: Filtering redundant and irrelevant information improves website usability for blind users. International Journal of Human-Computer Studies, 111, 23–35. 10.1016/j.ijhcs.2017.10.011

[bibr76-00187208261427867] Goldstein D. S. Bentho O. Park M. Y. Sharabi Y. (2011). Low‐frequency power of heart rate variability is not a measure of cardiac sympathetic tone but may be a measure of modulation of cardiac autonomic outflows by baroreflexes. Experimental Physiology, 96(12), 1255–1261. 10.1113/expphysiol.2010.056259 21890520 PMC3224799

[bibr77-00187208261427867] Grimes G. M. Valacich J. S. (2015). Mind over mouse: The effect of cognitive load on mouse movement behavior. In 2015 International Conference on Information Systems: Exploring the Information Frontier, ICIS 2015 (pp. 1–13). Association for Information Systems.

[bibr78-00187208261427867] Guerberof Arenas A. Moorkens J. O’Brien S. (2021). The impact of translation modality on user experience: An eyetracking study of the Microsoft Word user interface. Machine Translation, 35(2), 205–237. 10.1007/s10590-021-09267-z 34776636 PMC8550651

[bibr79-00187208261427867] Gupta K. Hajika R. Pai Y. S. Duenser A. Lochner M. Billinghurst M. (2019, November). In AI we trust: Investigating the relationship between biosignals, trust and cognitive load in VR. In 25th ACM symposium on virtual reality software and technology (pp. 1–10). Association for Computing Machinery.

[bibr80-00187208261427867] Gupta V. Kendre T. P. Reddy T. K. Arora V. (2022, May). Comparative performance analysis of scalp EEG and ear EEG based P300 ambulatory brain-computer interfaces using Riemannian geometry and convolutional neural networks. In 2022 National Conference on Communications (NCC) (pp. 314–319). IEEE.

[bibr81-00187208261427867] Guran A. M. Cojocar G. S. Dioşan L. (2020). A step towards preschoolers’ satisfaction assessment support by facial expression emotions identification. Procedia Computer Science, 176, 632–641. 10.1016/j.procs.2020.08.065

[bibr82-00187208261427867] Gwizdka J. (2010, August). Using Stroop task to assess cognitive load. In Proceedings of the 28th annual European conference on cognitive ergonomics (pp. 219–222). ACM.

[bibr83-00187208261427867] Haji F. A. Rojas D. Childs R. Ribaupierre S. Dubrowski A. (2015). Measuring cognitive load: Performance, mental effort and simulation task complexity. Medical Education, 49(8), 815–827. 10.1111/medu.12773 26152493

[bibr84-00187208261427867] Hart S. G. Staveland L. E. (1988). Development of NASA-TLX (Task Load Index): Results of empirical and theoretical research. Advances in Psychology, 52, 139–183. 10.1016/S0166-4115(08)62386-9

[bibr304-00187208261427867] Hess E. H. Polt J. M. (1964). Pupil size in relation to mental activity during simple problem-solving. Science, 143(3611), 1190–1192. 10.1126/science.143.3611.1190 17833905

[bibr85-00187208261427867] Hibbeln M. Jenkins J. L. Schneider C. Valacich J. S. Weinmann M. (2014, January). Investigating the effect of insurance fraud on mouse usage in human-computer interactions. In 35th international conference on information systems: Building a better world through information systems, ICIS 2014.

[bibr86-00187208261427867] Hirshfield L. M. Gulotta R. Hirshfield S. H. Hincks S. W. Russell M. Ward R. Williams T. Jacob R. J. K. (2011). This is your brain on interfaces: Enhancing usability testing with functional near-infrared spectroscopy. In Proceedings of the 29th ACM SIGCHI Conference on Human Factors in Computing Systems (pp. 373–382). ACM. 10.1145/1978942.1978996

[bibr87-00187208261427867] Hjortskov N. Rissén D. Blangsted A. K. Fallentin N. Lundberg U. Søgaard K. (2004). The effect of mental stress on heart rate variability and blood pressure during computer work. European Journal of Applied Physiology, 92(1), 84–89. 10.1007/s00421-004-1055-z 14991326

[bibr88-00187208261427867] Hocquet S. Ramel J. Y. Cardot H. (2004). *Users authentication by a study of human computer interactions* (Technical report, Proceedings of the Eighth Annual Doctoral Meeting on Health, Science and Technology). Université François-Rabelais, Tours, France.

[bibr89-00187208261427867] Holden R. J. Campbell N. L. Abebe E. Clark D. O. Ferguson D. Bodke K. Callahan C. M. Brain Health Patient Safety Laboratory . (2020). Usability and feasibility of consumer-facing technology to reduce unsafe medication use by older adults. Research in Social and Administrative Pharmacy, 16(1), 54–61. 10.1016/j.sapharm.2019.02.011 30853507 PMC6710164

[bibr90-00187208261427867] Holmqvist K. Nyström M. Andersson R. Dewhurst R. Jarodzka H. Van de Weijer J. (2011). Eye tracking: A comprehensive guide to methods and measures. Oxford University Press.

[bibr91-00187208261427867] Hu P. J. H. Ma P. C. Chau P. Y. (1999). Evaluation of user interface designs for information retrieval systems: A computer-based experiment. Decision Support Systems, 27(1-2), 125–143. 10.1016/s0167-9236(99)00040-8

[bibr92-00187208261427867] Huang T. Zhang J. (2022, June). Study on experience design of elderly online learning interface based on cognitive load. In Human-computer interaction. User experience and behavior: Thematic area, HCI 2022, held as part of the 24th HCI international conference, HCII 2022, virtual event, June 26–July 1, 2022, proceedings, part III (pp. 70–86). Springer International Publishing.

[bibr93-00187208261427867] Huang W. Eades P. Hong S. H. (2009). Measuring effectiveness of graph visualizations: A cognitive load perspective. Information Visualization, 8(3), 139–152. 10.1057/ivs.2009.10

[bibr94-00187208261427867] Hussain M. S. Calvo R. A. Chen F. (2014). Automatic cognitive load detection from face, physiology, task performance and fusion during affective interference. Interacting with Computers, 26(3), 256–268. 10.1093/iwc/iwt032

[bibr95-00187208261427867] Huttunen K. Keränen H. Väyrynen E. Pääkkönen R. Leino T. (2011). Effect of cognitive load on speech prosody in aviation: Evidence from military simulator flights. Applied Ergonomics, 42(2), 348–357. 10.1016/j.apergo.2010.08.005 20832770

[bibr96-00187208261427867] Imotions . (2021). The iMotions EDA/GSR module. https://imotions.com/biosensor/gsr-galvanic-skin-response-eda-electrodermal-activity/

[bibr97-00187208261427867] Innes R. J. Evans N. J. Howard Z. L. Eidels A. Brown S. D. (2021). A broader application of the detection response task to cognitive tasks and online environments. Human Factors, 63(5), 896–909. 10.1177/0018720820936800 32749155

[bibr98-00187208261427867] Jetter H. C. Reiterer H. Geyer F. (2014). Blended interaction: Understanding natural human–computer interaction in post-WIMP interactive spaces. Personal and Ubiquitous Computing, 18(5), 1139–1158. 10.1007/s00779-013-0725-4

[bibr99-00187208261427867] Jiang M. Liu S. Feng Q. Gao J. Zhang Q. (2018). Usability study of the user-interface of intensive care ventilators based on user test and eye-tracking signals. Medical Science Monitor: International Medical Journal of Experimental and Clinical Research, 24, 6617–6629. 10.12659/MSM.909933 30232319 PMC6161566

[bibr100-00187208261427867] Jin P. (2012). Redundancy effect. In Encyclopedia of the sciences of learning (pp. 2787–2788). Springer US.

[bibr101-00187208261427867] Johanssen J. O. Bernius J. P. Bruegge B. (2019, May). Toward usability problem identification based on user emotions derived from facial expressions. In 2019 IEEE/ACM 4th international workshop on emotion awareness in Software Engineering (SEmotion) (pp. 1–7). IEEE.

[bibr305-00187208261427867] Joseph A. W. Murugesh R. (2020). Potential eye tracking metrics and indicators to measure cognitive load in humancomputer interaction research. *Journal of Scientific Research*, 64(1), 268–275. 10.37398/jsr.2020.640137

[bibr102-00187208261427867] Jorgensen Z. Yu T. (2011, March). On mouse dynamics as a behavioral biometric for authentication. In Proceedings of the 6th ACM symposium on information, computer and communications security (pp. 476–482). Association for Computing Machinery.

[bibr104-00187208261427867] Just M. Carpenter P. Keller T. Emery L. Zajac H. Thulborn K. (2001). Interdependence of nonoverlapping cortical systems in dual cognitive tasks. NeuroImage, 14(2), 417–426. 10.1006/nimg.2001.0826 11467915

[bibr105-00187208261427867] Kalyuga S. Chandler P. Sweller J. (1999). Managing split-attention and redundancy in multimedia instruction. Applied Cognitive Psychology, 13(4), 351–371. 10.1002/(sici)1099-0720(199908)13:43.0.co;2-6

[bibr106-00187208261427867] Karim H. Schmidt B. Dart D. Beluk N. Huppert T. (2012). Functional near-infrared spectroscopy (fNIRS) of brain function during active balancing using a video game system. Gait & Posture, 35(3), 367–372. 10.1016/j.gaitpost.2011.10.007 22078300 PMC3294084

[bibr107-00187208261427867] Keim D. Andrienko G. Fekete J. D. Görg C. Kohlhammer J. Melançon G. (2008). Visual analytics: Definition, process, and challenges. In Information visualization (pp. 154–175). Springer.

[bibr108-00187208261427867] Keskin M. Ooms K. Dogru A. O. De Maeyer P. (2020). Exploring the cognitive load of expert and novice map users using EEG and eye tracking. ISPRS International Journal of Geo-Information, 9(7), 429. 10.3390/ijgi9070429

[bibr110-00187208261427867] Kettebekov S. (2004, October). Exploiting prosodic structuring of coverbal gesticulation. [Paper presented]. ICMI’04: 6th international conference on multimodal interfaces, State College, PA.

[bibr111-00187208261427867] Khawaja M. A. Chen F. Marcus N. (2012). Analysis of collaborative communication for linguistic cues of cognitive load. Human Factors, 54(4), 518–529. 10.1177/0018720811431258 22908676

[bibr112-00187208261427867] Khawaja M. A. Chen F. Marcus N. (2014). Measuring cognitive load using linguistic features: Implications for usability evaluation and adaptive interaction design. International Journal of Human-Computer Interaction, 30(5), 343–368. 10.1080/10447318.2013.860579

[bibr113-00187208261427867] Khawaja M. A. Ruiz N. Chen F. (2007). Potential speech features for cognitive load measurement. In Proceedings of the 19th Australasian conference on computer human interaction: Entertaining user interfaces (pp. 57–60). Association for Computing Machinery.

[bibr114-00187208261427867] Khawaji A. Chen F. Zhou J. Marcus N. (2014, December). Trust and cognitive load in the text-chat environment: The role of mouse movement. In Proceedings of the 26th Australian computer-human interaction conference on designing futures: The future of design (pp. 324–327). Association for Computing Machinery.

[bibr115-00187208261427867] King A. (2019). *Cognitive load and its impact on usage of email applications* (Bachelor’s thesis, Umeå University, Faculty of Social Sciences, Department of Psychology). Umeå University. https://www.diva-portal.org/smash/record.jsf?pid=diva2%3A1333121

[bibr116-00187208261427867] Kitabata M. Inazumi Y. Misawa T. Horita Y. Sugimoto O. Naito S. (2017, October). Can brain activity be used to evaluate the usability of smartphone devices? In 2017 IEEE 6th Global Conference on Consumer Electronics (GCCE) (pp. 1–2). IEEE.

[bibr117-00187208261427867] Kitchenham B. A. Charters S. (2007). Guidelines for performing systematic 1302 literature reviews in software engineering. EBSE.

[bibr118-00187208261427867] Klingner J. Kumar R. Hanrahan P. (2008, March). Measuring the task-evoked pupillary response with a remote eye tracker. In Proceedings of the 2008 symposium on eye tracking research & applications (pp. 69–72). Association for Computing Machinery.

[bibr119-00187208261427867] Koć-Januchta M. M. Schönborn K. J. Roehrig C. Chaudhri V. K. Tibell L. A. Heller H. C. (2022). “Connecting concepts helps put main ideas together”: Cognitive load and usability in learning biology with an AI-enriched textbook. International Journal of Educational Technology in Higher Education, 19(1), 11. 10.1186/s41239-021-00317-3

[bibr120-00187208261427867] Kortum P. Acemyan C. Z. (2016). The relationship between user mouse-based performance and subjective usability assessments. Proceedings of the Human Factors and Ergonomics Society - Annual Meeting, 60(1), 1174–1178. 10.1177/1541931213601275

[bibr121-00187208261427867] Kosch T. Karolus J. Zagermann J. Reiterer H. Schmidt A. Woźniak P. W. (2023). A survey on measuring cognitive workload in human-computer interaction. ACM Computing Surveys.

[bibr122-00187208261427867] Lai C. McMahan R. P. (2020, November). The cognitive load and usability of three walking metaphors for consumer virtual reality. In 2020 IEEE International Symposium on Mixed and Augmented Reality (ISMAR) (pp. 627–638). IEEE.

[bibr123-00187208261427867] Lamb R. Antonenko P. Etopio E. Seccia A. (2018). Comparison of virtual reality and hands on activities in science education via functional near infrared spectroscopy. Computers & Education, 124, 14–26. 10.1016/j.compedu.2018.05.014

[bibr124-00187208261427867] Lehmann J. A. M. Hamm V. Seufert T. (2019). The influence of background music on learners with varying extraversion: Seductive detail or beneficial effect? Applied Cognitive Psychology, 33(1), 85–94. 10.1002/acp.3509

[bibr125-00187208261427867] Lennon C. Burdick H. (2004). The Lexile framework as an approach for reading measurement and success. Electronic publication on. https://www.lexile.com/

[bibr126-00187208261427867] Li X. Zheng C. Pan Z. Huang Z. Niu Y. Wang P. Geng W. (2024). Comparative study on 2D and 3D user interface for eliminating cognitive loads in augmented reality repetitive tasks. International Journal of Human-Computer Interaction, 40(23), 8008–8024. 10.1080/10447318.2023.2276526

[bibr127-00187208261427867] Liu Y. Zheng B. Zhou H. (2019). Measuring the difficulty of text translation: The combination of text-focused and translator-oriented approaches. Target. International Journal of Translation Studies, 31(1), 125–149. 10.1075/target.18036.zhe

[bibr128-00187208261427867] Lively E. Pisoni D. B. Summers W. V. Bernacki R. (1993). Effects of cognitive workload on speech production: Acoustic analyses and perceptual consequences. Journal of the Acoustical Society of America, 93, 2962–2973. 10.1121/1.405815 8315159 PMC3499954

[bibr129-00187208261427867] Machado A. Oliveira A. Jácome C. Pereira M. Moreira J. Rodrigues J. Marques A. Jesus L. M. T. (2018). Usability of Computerized Lung Auscultation–Sound Software (CLASS) for learning pulmonary auscultation. Medical, & Biological Engineering & Computing, 56(4), 623–633. 10.1007/s11517-017-1697-8 28840490

[bibr130-00187208261427867] Majooni A. Akhavan A. Offenhuber D. (2018, July). An Eye-tracking study on usability and efficiency of blackboard platform. In International conference on applied human factors and ergonomics (pp. 281–289). Springer.

[bibr131-00187208261427867] Mandryk R. L. Atkins M. S. Inkpen K. M. (2006, April). A continuous and objective evaluation of emotional experience with interactive play environments. In Proceedings of the SIGCHI conference on human factors in computing systems (pp. 1027–1036). Association for Computing Machinery.

[bibr132-00187208261427867] Marcus N. Cooper M. Sweller J. (1996). Understand instructions. Journal of Educational Psychology, 88(1), 49–63. 10.1037//0022-0663.88.1.49

[bibr133-00187208261427867] Martin S. (2014). Measuring cognitive load and cognition: Metrics for technologyenhanced learning. Educational Research and Evaluation, 20(7-8), 592–621. 10.1080/13803611.2014.997140

[bibr134-00187208261427867] Martini F. Bartholomew E. (2001). Essentials of anatomy & physiology (p. 263). Benjamin Cummings.

[bibr135-00187208261427867] Mayer R. E. (2001). Multi-media learning. Cambridge University Press.

[bibr136-00187208261427867] Mazur L. M. Mosaly P. R. Moore C. Marks L. (2019). Association of the usability of electronic health records with cognitive workload and performance levels among physicians. JAMA Network Open, 2(4), Article e191709. 10.1001/jamanetworkopen.2019.1709 30951160 PMC6450327

[bibr308-00187208261427867] Messick S. (1995a). Standards of validity and the validity of standards in performance assessment. *Educational Measurement: Issues and Practice*, 14(4), 5–8. 10.1111/j.1745-3992.1995.tb00881.x

[bibr309-00187208261427867] Messick S. (1995b). Validity of psychological assessment: Validation of inferences from persons’ responses and performances as scientific inquiry into score meaning. *American Psychologist*, 50(9), 741–749. 10.1037/0003-066X.50.9.741

[bibr310-00187208261427867] Miller G. A. (1956). *Information and memory. Scientific American*, 195(2), 42–46. 10.1038/scientificamerican0856-42

[bibr137-00187208261427867] Miyake Y. Onishi Y. Poppel E. (2004). Two types of anticipation in synchronization tapping. Acta Neurobiologiae Experimentalis, 64(3), 415–426. 10.55782/ane-2004-1524 15283483

[bibr138-00187208261427867] Müller K. R. Tangermann M. Dornhege G. Krauledat M. Curio G. Blankertz B. (2008). Machine learning for real-time single-trial EEG-analysis: From brain–computer interfacing to mental state monitoring. Journal of Neuroscience Methods, 167(1), 82–90. 10.1016/j.jneumeth.2007.09.022 18031824

[bibr139-00187208261427867] Na K. (2021). The effects of cognitive load on query reformulation: Mental demand, temporal demand and frustration. Aslib Journal of Information Management, 73(3), 436–453. 10.1108/AJIM-07-2020-0206

[bibr140-00187208261427867] Nagy V. Kovács G. Földesi P. Kurhan D. Sysyn M. Szalai S. Fischer S. (2023). Testing road vehicle user interfaces concerning the driver’s cognitive load. Infrastructure, 8(3), 49. 10.3390/infrastructures8030049

[bibr141-00187208261427867] Nakasone A. Prendinger H. Ishizuka M. (2005, September). Emotion recognition from electromyography and skin conductance. In Proceedings of the 5th international workshop on biosignal interpretation (pp. 219–222). Tokyo, Japan.

[bibr142-00187208261427867] Nielsen J. (1994). Usability engineering. Morgan Kaufmann.

[bibr143-00187208261427867] Nourbakhsh N. Chen F. Wang Y. Calvo R. A. (2017). Detecting users’ cognitive load by galvanic skin response with affective interference. ACM Transactions on Interactive Intelligent Systems (TiiS), 7(3), 1–20. 10.1145/2960413

[bibr144-00187208261427867] Olive T. (2004). Working memory in writing: Empirical evidence from the dual-task technique. European Psychologist, 9(1), 32–42. 10.1027/1016-9040.9.1.32

[bibr145-00187208261427867] Oviatt S. (2006, October). Human-centered design meets cognitive load theory: Designing interfaces that help people think. In Proceedings of the 14th ACM international conference on multimedia (pp. 871–880). Association for Computing Machinery.

[bibr146-00187208261427867] Paas F. Ayres P. Pachman M. (2008). Assessment of cognitive load in multimedia learning. In Recent innovations in educational technology that facilitate student learning (pp. 11–35). Information Age Publishing Inc.

[bibr147-00187208261427867] Paas F. Renkl A. Sweller J. (2003). Cognitive load theory and instructional design: Recent developments. Educational Psychologist, 38(1), 1–4. 10.1207/s15326985ep3801_1

[bibr148-00187208261427867] Paas F. Renkl A. Sweller J. (2004). Cognitive load theory: Instructional implications of the interaction between information structures and cognitive architecture. Instructional Science, 32(1), 1–8. 10.1023/B:TRUC.0000021806.17516.d0

[bibr149-00187208261427867] Paas F. Tuovinen J. E. Tabbers H. Van Gerven P. W. (2016). Cognitive load measurement as a means to advance cognitive load theory. In Educational psychologist (pp. 63–71). Routledge.

[bibr150-00187208261427867] Paas F. van Merriënboer J. J. G. Adam J. J. (1994). Measurement of cognitive load in instructional research. Perceptual and Motor Skills, 79(1 Pt 2), 419–430. 10.2466/pms.1994.79.1.419 7808878

[bibr151-00187208261427867] Paas F. G. (1992). Training strategies for attaining transfer of problem-solving skill in statistics: A cognitive-load approach. Journal of Educational Psychology, 84(4), 429–434. 10.1037/0022-0663.84.4.429

[bibr152-00187208261427867] Pachunka E. (2018). Natural-setting PHR usability evaluation using eye tracking and NASA TLX to measure cognitive load of patients. [Doctoral dissertation, University of Nebraska at Omaha].

[bibr153-00187208261427867] Pachunka E. Windle J. Schuetzler R. Fruhling A. (2019, January). *Natural-setting PHR usability evaluation using the NASA TLX to measure cognitive load of patients. In Proceedings of the 52nd Hawaii International Conference on System Sciences* (pp. 1–10). Grand Wailea, Maui, HI, USA, January 8–11, 2019. Honolulu, HI: Hawaii International Conference on System Sciences. Retrieved from https://scholarspace.manoa.hawaii.edu/items/586f7fd9-1f34-484e-aa7c-2bec42dae642

[bibr154-00187208261427867] Padilla L. M. Castro S. C. Quinan P. S. Ruginski I. T. Creem-Regehr S. H. (2019). Toward objective evaluation of working memory in visualizations: A case study using pupillometry and a dual-task paradigm. IEEE Transactions on Visualization and Computer Graphics, 26(1), 332–342. 10.1109/TVCG.2019.2934286 31425092

[bibr155-00187208261427867] Page M. J. McKenzie J. E. Bossuyt P. M. Boutron I. Hoffmann T. C. Mulrow C. D. Moher D. Tetzlaff J. M. (2021). Updating guidance for reporting systematic reviews: Development of the PRISMA 2020 statement. Journal of Clinical Epidemiology, 134, 103–112. 10.1016/j.jclinepi.2021.02.003 33577987

[bibr156-00187208261427867] Pandian V. P. S. Suleri S. (2020). NASA-TLX web app: An online tool to analyse subjective workload. ArXiv Preprint arXiv:2001.09963.

[bibr157-00187208261427867] Park B. Brünken R. (2015). The rhythm method: A new method for measuring cognitive load—an experimental dual‐task study. Applied Cognitive Psychology, 29(2), 232–243. 10.1002/acp.3100

[bibr158-00187208261427867] Pecchinenda A. Petrucci M. (2016). Emotion unchained: Facial expression modulates gaze cueing under cognitive load. PLoS One, 11(12), Article e0168111. 10.1371/journal.pone.0168111 27959925 PMC5154609

[bibr159-00187208261427867] Pfleging B. Fekety D. K. Schmidt A. Kun A. L. (2016, May). A model relating pupil diameter to mental workload and lighting conditions. In Proceedings of the 2016 CHI conference on human factors in computing systems (pp. 5776–5788). Association for Computing Machinery.

[bibr160-00187208261427867] Plazak J. DiGiovanni D. A. Collins D. L. Kersten-Oertel M. (2019). Cognitive load associations when utilizing auditory display within image-guided neurosurgery. International Journal of Computer Assisted Radiology and Surgery, 14(8), 1431–1438. 10.1007/s11548-019-01970-w 30997635

[bibr161-00187208261427867] Pollack A. H. Pratt W. (2020). Association of health record visualizations with physicians’ cognitive load when prioritizing hospitalized patients. JAMA Network Open, 3(1), Article e1919301. 10.1001/jamanetworkopen.2019.19301 31940040 PMC6991320

[bibr162-00187208261427867] Porta M. Ricotti S. Perez C. J. (2012, April). Emotional e-learning through eye tracking. In Proceedings of the 2012 IEEE Global Engineering Education Conference (EDUCON) (pp. 1–6). IEEE.

[bibr163-00187208261427867] Pusara M. Brodley C. E. (2004, October). User re-authentication via mouse movements. In Proceedings of the 2004 ACM workshop on visualization and data mining for computer security (pp. 1–8). Association for Computing Machinery.

[bibr164-00187208261427867] Rafiqi S. Wangwiwattana C. Kim J. Fernandez E. Nair S. Larson E. C. (2015, July). PupilWare: Towards pervasive cognitive load measurement using commodity devices. In Proceedings of the 8th ACM international conference on PErvasive technologies related to assistive environments (pp. 1–8). Association for Computing Machinery.

[bibr165-00187208261427867] Rajavenkatanarayanan A. Nambiappan H. R. Kyrarini M. Makedon F. (2020, August). Towards a real-time cognitive load assessment system for industrial human-robot cooperation. In 2020 29th IEEE international conference on Robot and Human Interactive Communication (RO-MAN) (pp. 698–705). IEEE.

[bibr166-00187208261427867] Realpe-Muñoz P. Collazos C. A. Hurtado J. Granollers T. Muñoz-Arteaga J. Velasco-Medina J. (2018). *Eye tracking-based behavioral study of users using e-voting systems. Computer Standards & Interfaces, 55*, 182–195. 10.1016/j.csi.2017.08.004

[bibr168-00187208261427867] Reed W. M. Burton J. K. Kelly P. P. (1985). The effects of writing ability and mode of discourse on cognitive capacity engagement. Research in the Teaching of English, 19(3), 283–297. 10.58680/rte198515641

[bibr169-00187208261427867] Renshaw J. A. Finlay J. E. Tyfa D. Ward R. D. (2003). *Designing for visual influence: An eye tracking study of the usability of graphical management information.* In M. Rauterberg, C. Menozzi, & J. Wesson (Eds.), Human-Computer Interaction – INTERACT 2003 (pp. 144–151). IOS Press/IFIP.

[bibr170-00187208261427867] Rheem H. Verma V. Becker D. V. (2018). Use of mouse-tracking method to measure cognitive load. Proceedings of the Human Factors and Ergonomics Society - Annual Meeting, 62(1), 1982–1986. 10.1177/1541931218621449

[bibr171-00187208261427867] Rhodes J. K. Schindler D. Rao S. M. Venegas F. Bruzik E. T. Gabel W. Rudick R. A. Phillips G. A. Mullen C. C. Freiburger J. L. Mourany L. Reece C. Miller D. M. Bethoux F. Bermel R. A. Krupp L. B. Mowry E. M. Alberts J. (2019). Multiple sclerosis performance test: Technical development and usability. Advances in Therapy, 36(7), 1741–1755. 10.1007/s12325-019-00958-x 31054035 PMC6824297

[bibr172-00187208261427867] Richardson K. M. Fouquet S. D. Kerns E. McCulloh R. J. (2019). Impact of mobile device-based clinical decision support tool on guideline adherence and mental workload. Academic pediatrics, 19(7), 828–834. 10.1016/j.acap.2019.03.001 30853573 PMC6732014

[bibr173-00187208261427867] Rockman C. M. B. O’Shea C. K. W. Thomson R. H. Boyce M. W. Vey N. L. Geddes J. A. Colon C. E. T. (2020). Assessing cognitive load and usability for CEMA training using COBWebS.

[bibr174-00187208261427867] Rudmann D. S. McConkie G. W. Zheng X. S. (2003, November). Eyetracking in cognitive state detection for HCI. In Proceedings of the 5th international conference on multimodal interfaces (pp. 159–163). Association for Computing Machinery.

[bibr175-00187208261427867] Saadé R. G. Otrakji C. A. (2007). First impressions last a lifetime: Effect of interface type on disorientation and cognitive load. Computers in Human Behavior, 23(1), 525–535. 10.1016/j.chb.2004.10.035

[bibr176-00187208261427867] Schiessl M. Duda S. Thölke A. Fischer R. (2003). *Eye tracking and its application in usability and media research. MMI Interaktiv — Eye Tracking*, 1(06), 41–50. ISSN 1439-7854. Retrieved from https://dl.gi.de/items/87c38b31-343e-4018-ba18-7861c8356faa/full

[bibr177-00187208261427867] Schmutz P. Heinz S. Métrailler Y. Opwis K. (2009). *Cognitive load in eCommerce applications—Measurement and effects on user satisfaction. Advances in Human-Computer Interaction*, 2009, Article 121494. 10.1155/2009/121494

[bibr178-00187208261427867] Schomer D. L. Da Silva F. L. (2012). Niedermeyer’s electroencephalography: Basic principles, clinical applications, and related fields. Lippincott Williams & Wilkins.

[bibr179-00187208261427867] Schoor C. Bannert M. Brünken R. (2012). Role of dual task design when measuring cognitive load during multimedia learning. Educational Technology Research & Development, 60(5), 753–768. 10.1007/s11423-012-9251-8

[bibr180-00187208261427867] Schroeder N. Cenkci L. (2018). Spatial contiguity and spatial split-attention effects in multimedia learning environments: A meta-analysis. Educational Psychology Review, 30(3), 679–701. 10.1007/s10648-018-9435-9

[bibr181-00187208261427867] Sengupta K. Sun J. Menges R. Kumar C. Staab S. (2017, June). Analyzing the impact of cognitive load in evaluating gaze-based typing. In 2017 IEEE 30th international symposium on Computer-Based Medical Systems (CBMS) (pp. 787–792). IEEE.

[bibr182-00187208261427867] Sevcenko N. Appel T. Ninaus M. Moeller K. Gerjets P. (2023). Theory-based approach for assessing cognitive load during time-critical resource-managing human–computer interactions: An eye-tracking study. Journal on Multimodal User Interfaces, 17(1), 1–19. 10.1007/s12193-022-00398-y

[bibr183-00187208261427867] Sharp H. Preece J. Rogers Y. (2019). Interaction design: Beyond human-computer interaction. Jon Wiley & Sons Inc.

[bibr184-00187208261427867] Shelton B. Nesbitt K. Thorpe A. Eidels A. (2021). Gauging the utility of ambient displays by measuring cognitive load. Cognition, Technology & Work, 23(3), 459–480. 10.1007/s10111-020-00639-8

[bibr185-00187208261427867] Shi Y. Taib R. Ruiz N. Choi E. Chen F. (2007). Multimodal human-machine interface and user cognitive load measurement. IFAC Proceedings Volumes, 40(16), 200–205. 10.3182/20070904-3-kr-2922.00035

[bibr186-00187208261427867] Siegle G. J. J. Steinhauer S. R. R. Thase M. E. E. (2004). Pupillary assessment and computational modeling of the Stroop task in depression. International Journal of Psychophysiology, 52(1), 63–76. 10.1016/j.ijpsycho.2003.12.010 15003373

[bibr187-00187208261427867] Silva C. Vieira J. Campos J. C. Couto R. Ribeiro A. N. (2021). Development and validation of a descriptive cognitive model for predicting usability issues in a low-code development platform. Human Factors, 63(6), 1012–1032. 10.1177/0018720820920429 32442034

[bibr188-00187208261427867] Solhjoo S. Haigney M. C. McBee E. van Merrienboer J. J. Schuwirth L. Artino A. R. Durning S. J. Ratcliffe T. A. Lee H. D. (2019). Heart rate and heart rate variability correlate with clinical reasoning performance and self-reported measures of cognitive load. Scientific Reports, 9(1), 1–9. 10.1038/s41598-019-50280-3 31604964 PMC6789096

[bibr189-00187208261427867] Solovey E. T. Zec M. Garcia Perez E. A. Reimer B. Mehler B. (2014, April). Classifying driver workload using physiological and driving performance data: Two field studies. In Proceedings of the SIGCHI conference on human factors in computing systems (pp. 4057–4066). Association for Computing Machinery.

[bibr190-00187208261427867] Stojmenova K. Sodnik J. (2018). Detection-response task—uses and limitations. Sensors, 18(2), 594. 10.3390/s18020594 29443949 PMC5855461

[bibr191-00187208261427867] Sutton R. S. Barto A. G. (2018). Reinforcement learning: An introduction (2nd ed., Vol. 1, p. 25). MIT Press Cambridge.

[bibr192-00187208261427867] Suzuki Y. Wild F. Scanlon E. (2024). Measuring cognitive load in augmented reality with physiological methods: A systematic review. Journal of Computer Assisted Learning, 40(2), 375–393. 10.1111/jcal.12882

[bibr193-00187208261427867] Sweller J. (1988). Cognitive load during problem solving: Effects on learning. Cognitive Science, 12(2), 257–285. 10.1207/s15516709cog1202_4

[bibr194-00187208261427867] Sweller J. (1994). Cognitive load theory, learning difficulty, and instructional design. Learning and Instruction, 4(4), 295–312. 10.1016/0959-4752(94)90003-5

[bibr195-00187208261427867] Sweller J. (2010). Element interactivity and intrinsic, extraneous, and germane cognitive load. Educational Psychology Review, 22(2), 123–138. 10.1007/s10648-010-9128-5

[bibr196-00187208261427867] Sweller J. (2011). Cognitive load theory. In J. P. Mestre & B. H. Ross (Eds.), *Psychology of learning and motivation* (Vol. 55, pp. 37–76). Academic Press. 10.1016/B978-0-12-387691-1.00002-8

[bibr197-00187208261427867] Sweller J. (2025). An integrated human cognitive architecture. Educational Psychology Review, 37(4), 108. 10.1007/s10648-025-10089-1

[bibr198-00187208261427867] Sweller J. Ayres P. Kalyuga S. (2011). Cognitive load theory. Springer.

[bibr199-00187208261427867] Sweller J. van Merriënboer J. J. Paas F. (2019). Cognitive architecture and instructional design: 20 years later. Educational Psychology Review, 31(2), 261–292. 10.1007/s10648-019-09465-5

[bibr200-00187208261427867] Thüring M. Mahlke S. (2007). Usability, aesthetics and emotions in human-technology interaction. International Journal of Psychology, 42(4), 253–264. 10.1080/00207590701396674.

[bibr201-00187208261427867] Tuček D. C. Mount W. M. Abbass H. A. (2012, November). Neural and speech indicators of cognitive load for Sudoku game interfaces. In International conference on neural information processing (pp. 210–217). Springer.

[bibr202-00187208261427867] Van Merrienboer J. Sweller J. (2005). Cognitive load theory and complex learning: Recent developments and future directions. Educational Psychology Review, 17(2), 147–177. 10.1007/s10648-005-3951-0

[bibr203-00187208261427867] Van Mierlo C. M. Jarodzka H. Kirschner F. Kirschner P. A. (2012). Cognitive load theory in elearning. In Yan Z. (Ed.), Encyclopedia of cyber behavior (pp. 1178–1211). IGI Global.

[bibr204-00187208261427867] Van Nuland S. E. (2017). Examination and assessment of commercial anatomical e-learning tools: Software usability, dual-task paradigms and learning (Doctoral dissertation). The University of Western Ontario, Electronic Thesis and Dissertation Repository. https://ir.lib.uwo.ca/etd/4615/

[bibr205-00187208261427867] Veilleux M. Sénécal S. Demolin B. Bouvier F. Di Fabio M. L. Coursaris C. Léger P. M. (2020, July). Visualizing a user’s cognitive and emotional journeys: A Fintech case. In International conference on human-computer interaction (pp. 549–566). Springer.

[bibr206-00187208261427867] Wang Q. Yang S. Liu M. Cao Z. Ma Q. (2014). An eye-tracking study of website complexity from cognitive load perspective. Decision Support Systems, 62, 1–10. 10.1016/j.dss.2014.02.007

[bibr207-00187208261427867] Wang Y. Yu S. Ma N. Wang J. Hu Z. Liu Z. He J. (2020). Prediction of product design decision making: An investigation of eye movements and EEG features. Advanced Engineering Informatics, 45, 101095. 10.1016/j.aei.2020.101095

[bibr208-00187208261427867] Wenk N. Penalver-Andres J. Buetler K. A. Nef T. Müri R. M. Marchal-Crespo L. (2023). Effect of immersive visualization technologies on cognitive load, motivation, usability, and embodiment. Virtual Reality, 27(1), 307–331. 10.1007/s10055-021-00565-8 36915633 PMC9998603

[bibr209-00187208261427867] Wood C. Torkkola K. Kundalkar S. (2004). Using driver’s speech to detect cognitive workload . In 9th Conference speech and Computer (SPECOM 2004) (pp. 215–222). International Speech Communication Association.

[bibr210-00187208261427867] Wu C. H. Liu C. H. Huang Y. M. (2022). The exploration of continuous learning intention in STEAM education through attitude, motivation, and cognitive load. International Journal of STEM Education, 9(1), 1–22. 10.1186/s40594-022-00346-y

[bibr211-00187208261427867] Xiao Y. M. Wang Z. M. Wang M. Z. Lan Y. J. (2005). The appraisal of reliability and validity of subjective workload assessment technique and NASA-task load index. Zhonghua Lao Dong Wei Sheng Zhi Ye Bing Za Zhi, 23(3), 178–181. 10.3760/cma.j.issn.1001-9391.2005.03.007. 16124892

[bibr212-00187208261427867] Xu N. Guo G. Lai H. Chen H. (2018). Usability study of two in-Vehicle information systems using finger tracking and facial expression recognition technology. International Journal of Human-Computer Interaction, 34(11), 1032–1044. 10.1080/10447318.2017.1411674

[bibr213-00187208261427867] Yates N. Gough S. Brazil V. (2022). Self-assessment: With all its limitations, why are we still measuring and teaching it? Lessons from a scoping review. Medical Teacher, 44(11), 1296–1302. 10.1080/0142159X.2022.2093704 35786121

[bibr214-00187208261427867] Yin B. Chen F. Ruiz N. Ambikairajah E. (2008). Speech-based cognitive load monitoring system. In 2008 IEEE international conference on acoustics, speech and signal processing (pp. 2041–2044). IEEE. 10.1109/ICASSP.2008.4518041

[bibr215-00187208261427867] Zagermann J. Pfeil U. Reiterer H. (2016, October). Measuring cognitive load using eye tracking technology in visual computing. In Proceedings of the sixth workshop on beyond time and errors on novel evaluation methods for visualization (pp. 78–85). Association for Computing Machinery.

[bibr216-00187208261427867] Zardari B. A. Hussain Z. Arain A. A. Rizvi W. H. Vighio M. S. (2021). QUEST e-learning portal: Applying heuristic evaluation. Usability Testing and Eye Tracking, 20(3), 531–543. 10.1007/s10209-020-00774-z

[bibr217-00187208261427867] Zheng R. Z. (Ed.), (2017). Cognitive load measurement and application: A theoretical framework for meaningful research and practice. Routledge.

[bibr218-00187208261427867] Zhou T. Cha J. S. Gonzalez G. Wachs J. P. Sundaram C. P. Yu D. (2020). Multimodal physiological signals for workload prediction in robot-assisted surgery. ACM Transactions on Human-Robot Interaction (THRI), 9(2), 1–26. 10.1145/3368589

